# Home Scholarly Culture, Book Selection Reason, and Academic Performance: Pathways to Book Reading Interest among Secondary School Students

**DOI:** 10.3390/ejihpe11020034

**Published:** 2021-06-01

**Authors:** Quan-Hoang Vuong, Minh-Hoang Nguyen, Tam-Tri Le

**Affiliations:** 1 Centre for Interdisciplinary Social Research, Phenikaa University, Yen Nghia Ward, Ha Dong District, Hanoi 100803, Vietnam; hoang.vuongquan@phenikaa-uni.edu.vn; 2 A.I. for Social Data Lab (AISDL), Vuong & Associates, Hanoi 100000, Vietnam

**Keywords:** book reading, student academic performance, home scholarly culture, reading preferences, STEM learning

## Abstract

Although studies have explored the predictors of book reading interest among children, little is known about the underlying mechanism that helps children become interested in reading books. This study attempt to demonstrate: (1) how book-reading interest is driven by reasons for choosing books (recommendation or personal preference), (2) how students with high and low academic achievements are motivated by different thinking pathways, and (3) how home scholarly culture improves book-reading interest through such pathways. Using Bayesian analysis on a dataset of survey responses from 4966 Vietnamese secondary students (11–15 years old, sixth to ninth grade), we found: (i) Reading interest is positively associated with a book recommendation and parental book reading activities (parents read books to children); (ii) High-achieving students are more interested in reading books if they can choose those books according to personal preferences; (iii) Parental book reading activities can promote book reading interest through recommendations and also by understanding children’s personal preferences. We advocate a more personalized approach in educational policymaking, curriculum design, and home scholarly culture based on students’ abilities and perceptions.

## 1. Introduction

Book reading, throughout history, has always been an essential part of human civilization. Together with the advancement of modern technology, reading materials have also gained various new forms such as audio or digital books. Today, reading is still a powerful tool for learning regardless; without a doubt, it is a crucial element in education systems worldwide. The act of reading for acquiring knowledge and developing creativity is a fundamental aspect of the natural human development process in society [1]. Research has shown that parent–child book-reading activities enhance children’s reading skills, whereas discussions between parents and children about books have positive effects on children’s academic performance [2]. Interactions with new vocabularies along with reading comprehension practices also enhance children’s writing skills, which help them greatly in different environments [3]. Reading is beneficial for academic activities in both high and low-income environments across the world [4]. In brief, reading helps children acquire more information and increase their knowledge, academic skills, as well as overall social competence.

Reading is an indispensable tool in STEM (science, technology, engineering, and mathematics) education. STEM education has been a strong focus in educational systems worldwide to support the advancement of natural sciences and develop young generations of scientists, researchers, and engineers [5]. Reading STEM content is challenging in higher education [6], so growing book reading interest from a young age can be of great help. Students spending more time reading natural science books often score higher in STEM subjects [7]. Globally, specialized STEM education is often employed from the secondary school level, stressing the importance of encouraging children’s reading behaviors [8].

An effective factor for encouraging reading behaviors is home scholarly culture—creating literacy and a book-oriented environment for children at home. It has three major aspects: early home literacy activities, parental attitudes toward reading, and the number of books at home [9]. According to the data from the Programme for the International Assessment of Adult Competencies (PIAAC) between 2011 and 2015, those who were brought up in book-reading-oriented environments tend to perform better in studying and working [10]. Families with plenty of books and considering reading as a leisure activity create a better environment for children to study [11]. In-home scholarly culture, parents are the models, motivators, and facilitators who accompany children in reading activities, encourage them to read and provide them with various types of books [12]. In brief, parents hold a significant role in providing the optimal learning environment for their children, especially book-reading.

The present study focuses on Vietnamese children’s book reading behaviors. Based on the dataset of 4966 Vietnamese secondary students, the study aims to investigate the research questions below. The reasons why these research questions are proposed will be presented clearly in the next section.

(i) How is book-reading interest driven by reasons for choosing books (recommendations or personal preferences)?

(ii) Are students with high and low academic achievements motivated by different thinking pathways (recommendations or personal preferences)?

(iii) How does home scholarly culture improve book-reading interest among low- and high-achieving students through such pathways (recommendations or personal preferences)?

In the next section, a literature review of factors predicting reading motivations among students and the need for a new theoretical approach is provided. The third section explains the current study’s theoretical foundation using Mindsponge theory. Based on the theoretical foundation, we also propose three statistical models for answering the research questions in the third section. The current study’s materials, variables, and statistical analysis are described in the next section. The results obtained from Bayesian analyses are presented in the fifth section according to the statistical models proposed in the third section. In the final section, we discuss the findings’ values of this study along with their implications for educational policy as well as the study’s limitations.

## 2. Literature Review

Reading motivation is considered an essential component in creating and developing the reading capacity of students. It can be classified into many dimensions, including self-efficacy, intrinsic and extrinsic reading motives, and social aspects of reading [13]. Reading motivation has positive effects on oral reading frequency and reading comprehension [14]. Other studies also provide evidence supporting that children’s motivation is a strong predictor for reading comprehension [15,16,17]. Reading motivation and reading amount are positively associated with reading achievement, academic performance, and students’ world knowledge [18,19,20,21].

What are the factors that might improve or predict reading motivations in children? Various factors have been shown to generate effects on reading motivation among children in previous literature. Those factors could be grouped into two categories: (i) individual’s identities and traits (e.g., age, gender, and reading competence) and (ii) environmental characteristics (e.g., family culture, schooling environment, and social norms).

### 2.1. Individual’s Identities and Traits

Gender is a complex factor regarding reading motivation in children. However, some studies pointed out that girls tend to show a higher reading motivation level than boys. Boys were reported to be less motivated to read than girls, and that reading might be perceived as a feminine endeavor [22,23,24,25]. Besides, motivation for reading in male students was stated to decrease faster than in female students [26], reading self-concept and reading value in female students are better than in male students [22]; girls have an advantage over boys of the same reading level in constructed-response items [27]. However, in the relationship between reading motivation and reading amount or reading comprehension, gender does not have any significant influence [28].

Age is another factor affecting reading motivation. Studies of both Western and Eastern environments mentioned that reading motivation in students of lower grades is more positive than in those of higher grades [7,25,29,30], which means that generally, the motivation for reading in students decreases over time [31]. However, there is also the opposite situation: eighth-grade students were found to have more positive motivation on reading and higher reading self-efficacy than six-graders; additionally, eight-graders do not expect to be suggested reading materials, while six-graders need such support [32]. To explore deeper and find the underlying mechanism, we should examine more complex factors besides demographics.

Reading competence can improve reading motivation and vice versa. The relationship between reading motivation and reading performance was studied in both directions: high reading motivation is advantageous for students who want to achieve better reading proficiency [33,34]. On the other hand, students who gain satisfactory results in education have more reading motivation to enhance their knowledge [7] further. It is consistent with a previous study stating that reading motivation and self-concept of poor readers decline throughout the school year while reading motivation in good readers remains at a high level steadily [35].

### 2.2. Environmental Characteristics

Besides individual traits, the external environment (including family, school, and society) plays an important role in changing students’ reading motivation. The interactions between family and school are deemed particularly effective [36]. Family in general and home scholarly culture are the primary motivators from early adolescence in developing reading ability, maintaining a love of reading, and supporting children’s academic performance and future occupations [10,37]. One of the first steps of building a home scholarly culture can be reading stories to children before bed, which was found to be a strong factor for motivating children toward reading [38]. Parents’ level of knowledge also influences students’ reading attitudes and reading behaviors because parents with high qualifications understand the importance of reading and aim to create many opportunities for their children to participate in reading activities [39]. Good financial background also positively affects reading motivation in children [7]. On the contrary, being unable to access reading materials is a huge hindrance in building children’s interest in reading [40]. This argument is consistent with a study stating that students from small families with only one or two children have more motivation to read since their parents can spend more money and time caring for their reading behavior and interest [38].

While the family environment initiates and facilitates behaviors and motivations for reading in children, the school takes the role of maintaining and sustaining them, in which the autonomy-supportive, structured, and involved teacher behaviors are the essential elements [14,41]. However, teachers’ perceptions were found to have weaker effects on male students than on female students regarding reading motivation, self-concept, and reading task value [42]. Furthermore, a school library with a wide variety of books increases students’ reading motivation as they can find books they are interested in, especially for students with low financial capability [43]. The diversity of books at the school library also increases students’ motivation toward reading specialized books [7], which indirectly enhances subject-related knowledge and consequently academic performance.

Besides the collaboration between family and school, society’s collective attention also contributes significantly to the development of reading motivation and behavior. Reading activities such as reading competitions or storytelling contests enhance reading time and children’s reading interest [44]. Such competitions encourage students to read more actively, deeply and find reading materials independently [45]. In poor regions with limited access to knowledge sources, public libraries with diverse book types are crucial in motivating students to read [44].

### 2.3. The Need for a New Approach

While previous studies mentioned many factors on reading motivation and presented suggestions for parents and teachers to support better reading habits in children, there is a lack of clear demonstrations about the psychological mechanism of how book reading interest is enhanced or diminished in mind. Different thinking pathways related to book reading interest between high and low-achieving students might also be worth investigating. Such a psychological mechanism is complex in terms of information processing, especially when incorporating other social interactions, such as home scholarly culture. This requires a flexible and dynamic framework of cognition shifting. Therefore, we decided to use the Mindsponge theory as the theoretical foundation due to its characteristics described in the following section. This paper aims to fill the research gap in reading interest psychology, focusing on the difference between low and high-achieving students and the role of home scholarly culture.

## 3. Theoretical Foundation

This study uses the Mindsponge theory [46,47,48] as the theoretical foundation for explaining the mechanism of book-reading motivation in secondary school students. Mindsponge is a framework of how information from accessible sources in the environment is processed by the mind through a complex multi-filtering apparatus using cost-benefit judgments. Such judgments are based on external conditions (contexts) and internal conditions (personal beliefs and preferences from memory), determining whether the information is accepted or rejected. If the perceived benefit of a certain piece of information is deemed higher than its perceived cost, then the information is accepted, and vice versa. The degree of influence of the accepted information depends on the relative distance between their values and core beliefs. Accepted information which is deeply integrated into the mindset (core beliefs) can lead to subsequent changes in the filters toward later evaluations of related information (easier to accept or reject) by becoming new trusted references. This updating mechanism can cause a feedback loop that reinforces directional reaction toward similar information by gradually changing the mindset.

The Mindsponge theory is an effective framework for explaining the shift in cognition, potentially applicable for many different disciplines, especially in psychology. A similar application of this theory was used in a study for exploring the mechanism of suicidal ideation in terms of information processing, which led to a new approach in suicidal thought prevention research [49]. With that in mind, we apply the same principles to the targets of this study: the perceived value of book-reading regarding reading interest, source preference, and parental book reading.

In the present study, due to the structure of the related survey questions, information availability and accessibility are theoretically equally given. Therefore, we solely focus on the aspect of information’s value evaluation.

The principles of the mechanism of book-reading interest are presented as follows: (i) a child wants to read a book when he/she perceives the benefit of doing so as being greater than its perceived cost; (ii) when the reason for acquiring books aligns with the child’s belief(s), the perceived benefit (of reading) increases, and vice versa; (iii) such reason is either external (e.g., recommendation) or internal (e.g., personal preference) and the filters (trust evaluators) can also be influenced by interactions with specific information sources (e.g., parents reading books to children).

**Figure 1:**
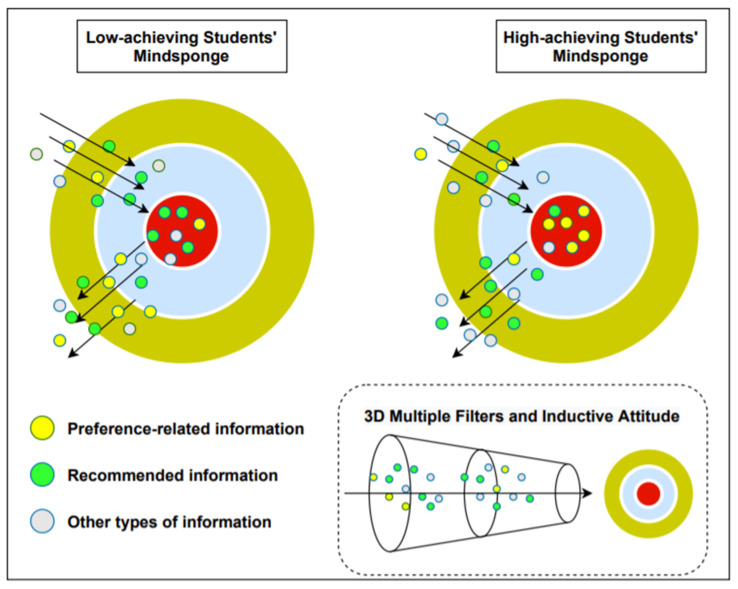
Mindsponge processes of acquiring information in low and high-achieving students.

The Mindsponge information-processing mechanism of low- and high-achieving students can be visualized in Figure 1. In Figure 1, the yellow area of the sponge represents the surrounding environment, the light blue area represents the buffer zone (where information is waiting to be evaluated), and the red area represents the mindset (core beliefs). The 3D multiple filters determine the acceptance and rejection of information through inductive attitudes. There are three types of information here: preference-related information (yellow dots), recommended information (green dots), and other types of information (gray dots). When any kind of information is in the mindset, it will influence the subsequent cost-benefit evaluation process of newly entering information. Further explanation of the visualization will be provided along with statistical results in the Discussion section.

Based on the theoretical reasoning above, we present the research objectives as follows.

**Step 1.** We want to know if the external or internal initiation in acquiring information helps increase reading interest and whether there is any difference in academic performance. We examine the effect of the reason for choosing a preferred type of book and its interaction with STEM performance on reading interest. The reason is either recommendation or personal preference. We use two opposite groups of low and high-achieving students (see data description in the Methodology section). Model 1 is as follows.

(1) Readbook ~ α+Reason_PR+Reason_PR∗HighAPS+Reason_PR∗LowAPS

**Step 2.** Following the Mindsponge mechanism of information’s value evaluation, we expand the information processing to social interactions; specifically, we want to know how family scholarly culture influences the perception of information sources regarding choosing books. We examine the effect of parental book reading (parents reading stories for their children) and its interactions with academic performance on the reason for choosing a preferred type of book. The results in Step 1 and Step 2 should be consistent regarding the proposed mechanism.

(2) Reason_PR ~ α+Readstory+Readstory∗HighAPS+Readstory∗LowAPS.

**Step 3.** Finally, to have a complete view of this mechanism, we examine the effect of parental book reading on book-reading interest and its interactions with each group of students based on STEM performance. This step can provide deeper insights into the information evaluation process, particularly through interactions with parents. We use the following model for Step 3.

(3) Readbook ~ α+Readstory+Readstory∗HighAPS+Readstory∗LowAPS.

## 4. Methodology

### 4.1. Material

This study employs the dataset from the survey on reading habits and preference of junior high school students in Vietnam designed by Vuong and Associates office. The data collection was conducted in two periods: from December 2017 to January 2018 and from February until July 2018. There were 4966 students, 11–15 years old (from 6th grade to 9th grade), from 16 secondary schools in Ninh Binh Province responding to the survey. All steps of the data collection followed the institutions’ ethical code. The questionnaire was explained clearly to all students before they responded. Among respondents, the percentages of male and female students were 49.36% and 49.62%, respectively; and the percentages of students in all four grades were quite similar: 24.90%, 24.04%, 23.86%, and 25.51% from sixth to ninth grade, respectively. The complete description and validation of the dataset can be viewed in the article “A data collection on secondary school students’ STEM performance and reading practices in an emerging country” [50]. This is an open dataset, which is cost-effective and transparent [51]. Supplementary materials are openly available on the OSF server, DOI: 10.17605/OSF.IO/UCZDW.

### 4.2. Variables

The variables are described in Table 1.

### 4.3. Statistical Analysis

This paper uses Bayesian analysis with the Hamiltonian MCMC technique (Markov Chain Monte Carlo). Bayesian inference offers three main advantages presented as follows. First, the MCMC algorithms iteratively generate a large number of samples [52,53], which helps make an accurate estimation for the posterior distribution of any parameters, and thus fits with Mindsponge characteristics of (i) cost-benefit judgments influenced by both external contexts and internal preferences, and (ii) the multiplex nature of information processing creating non-linear causal nexus. Second, given the dynamics, multiplexity, and updating manner of the Mindsponge processes, the number of control parameters might be too large to decrease the model’s residuals. However, Bayesian inference treats all properties as probabilities [54], allowing flexible and reliable examinations of certain targets, such as those in this study. Third, the Bayesian belief updating process [55] fundamentally aligns with updating information evaluation in the Mindsponge theory. Regarding the complexity of psychological processes and social issues, this characteristic also helps against the reproducibility crisis in psychological research [56]. The effectiveness of utilizing Bayesian analysis for investigating Mindsponge psychological processes was shown in a previous study [49].

To conduct the analysis, based on the dataset, we created three new variables *LowAPS*, *HighAPS*, and *Reason_PR*, along with two existing ones *Readbook* and *Readstory*. The data were analyzed using R software (version 4.0.2) with the *bayesvl* package [57,58,59]. We used Bayesian MCMC estimation with 5000 iterations (2000 are warm-up iterations), four Markov chains, and four cores; we also used the Pareto smoothed importance-sampling leave-one-out cross-validation (PSIS-LOO) approach [60] to check for goodness-of-fit. Since this is the first study of its scope and method, all estimations’ priors were set as uninformative [61].

## 5. Results

### 5.1. Model 1

The purpose of Model 1 is to examine whether recommendation or self-preference (*Reason_PR*) might improve children’s reading interest (*Readbook*) and how children’s academic performance might amplify or moderate the effect (*HighAPS_Reason_PR* and *LowAPS_Reason_PR*).

**Figure A2:**
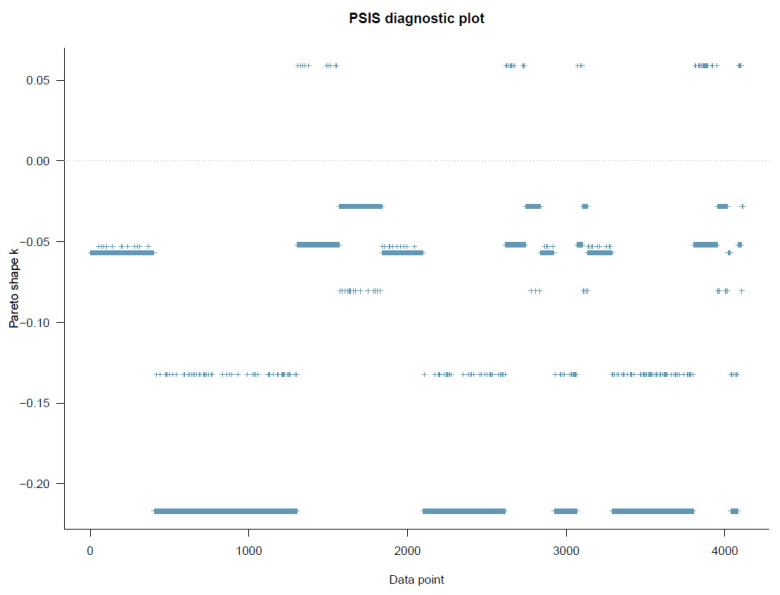
Model 1’s PSIS diagnostic plot.

For estimating Model 1, we employed Bayesian MCMC simulation with 5000 iterations, 2000 warm-up iterations, and four Markov chains. All the priors of the model’s coefficients were set as ‘uninformative’, which is equivalent to a normal distribution with mean = 0 and standard deviation = 10. Before diagnosing the convergence of the model, the PSIS diagnostic plot was visualized for checking the goodness-of-fit of Model 1 against the data. As the Pareto k estimates are all good (k < 0.5), it can be said that the model fits the data reasonably well (see Appendix A, Figure A2).

**Figure A3:**
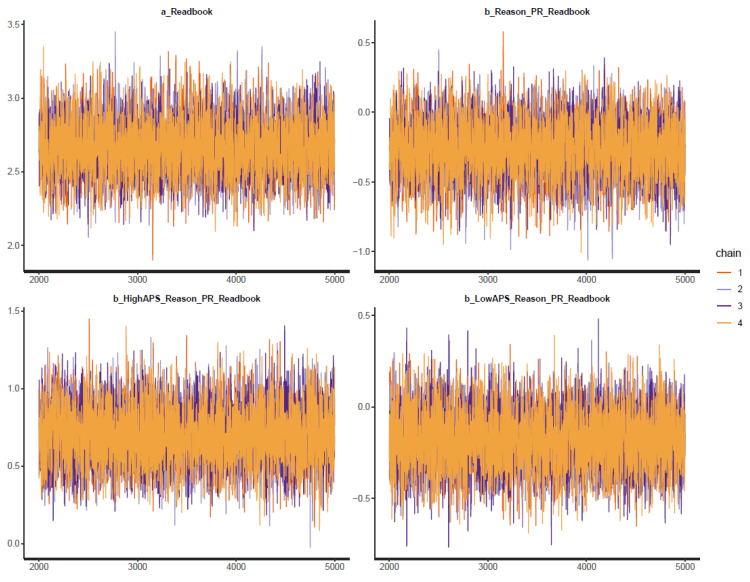
Trace plots for Model 1’s posterior coefficients.

**Figure A4:**
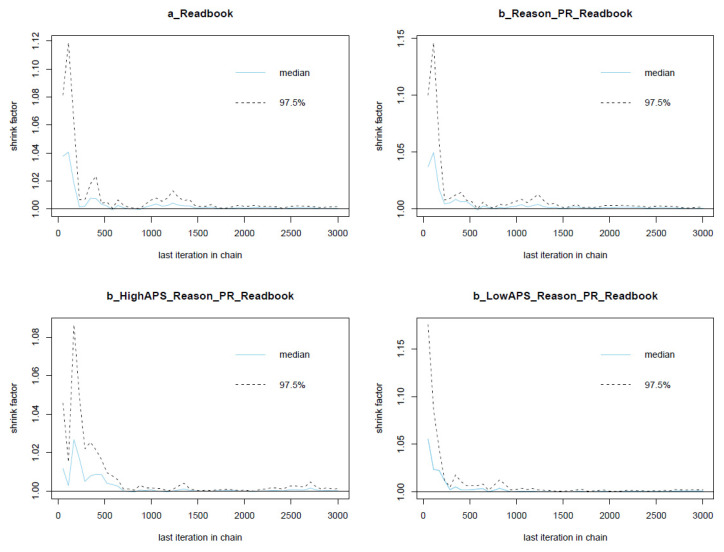
Gelman plots for Model 1’s posterior coefficients.

**Figure A5:**
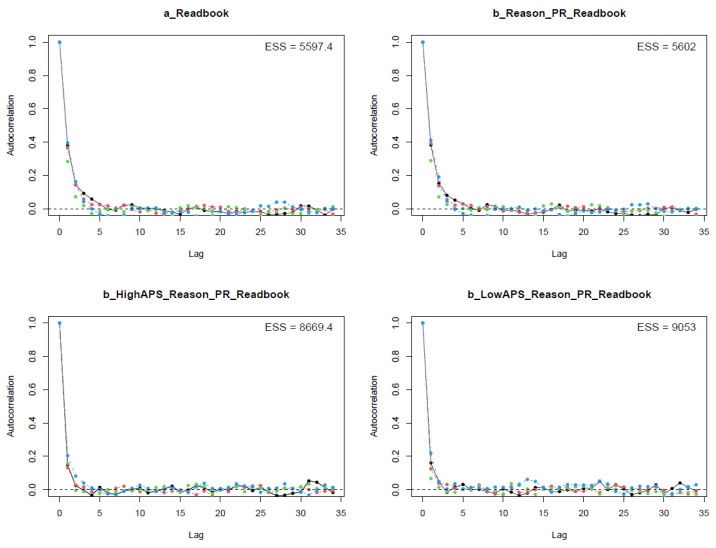
Autocorrelation plots for Model 1’s posterior coefficients.

Model 1 shows a good convergence, which can be interpreted using two standard diagnostics of MCMC: n_eff’s values (effective sample size) being greater than 1000 and Rhat’s values (Gelman shrink factor) equaling one (see Table 2). In addition, the convergence of Model 1 was also confirmed visually using trace plots, Gelman plots, and autocorrelation plots (see Appendix A, Figure A3, Figure A4 and Figure A5).

Each trace plot of the four Markov chains fluctuates around a central equilibrium (see Appendix A, Figure A3). In general, no divergent chains are found after the warm-up iterations, suggesting the model’s good convergence.

The Gelman and autocorrelation plots were also produced to check whether the random process has the Markov property (or convergence in distribution is achieved). On the one hand, according to Gelman and Rubin (1992), convergence is achieved when there is almost no difference between variance between chains and variance within chains. In other words, the model is convergent when the shrink factors reduce to one during the warm-up iterations (see Appendix A, Figure A4). On the other hand, the Markov property is only held if the MCMC-simulated samples are not autocorrelated (or memoryless during the stochastic simulation process). Visually, the autocorrelations are eliminated quickly after certain finite steps (see Appendix A, Figure A5).

As shown in Table 2, students who chose a preferred type of book by being recommended are more likely to be interested in reading books than those who chose a preferred type of book by personal preference ($\mu_{Reason\_PR}$ = −0.26 and $\sigma_{Reason\_PR}$ = 0.20). However, when adding the interaction with high APS, the influence of recommendation on reading interest is moderated ($\mu_{Reason\_PR*HighAPS}$ = 0.70 and $\sigma_{Reason\_PR*HighAPS}$ = 0.18). Meanwhile, the interaction between low APS and reason to choose a preferred type of book results in a negative association with book reading interest ($\mu_{Reason\_PR*LowAPS}$μ = −0.17; $\sigma_{Reason\_PR*LowAPS}$ = 0.16). Thus, it is plausible to say that the impact of the recommendation on reading interest might be amplified among students with a low APS.

**Figure 2:**
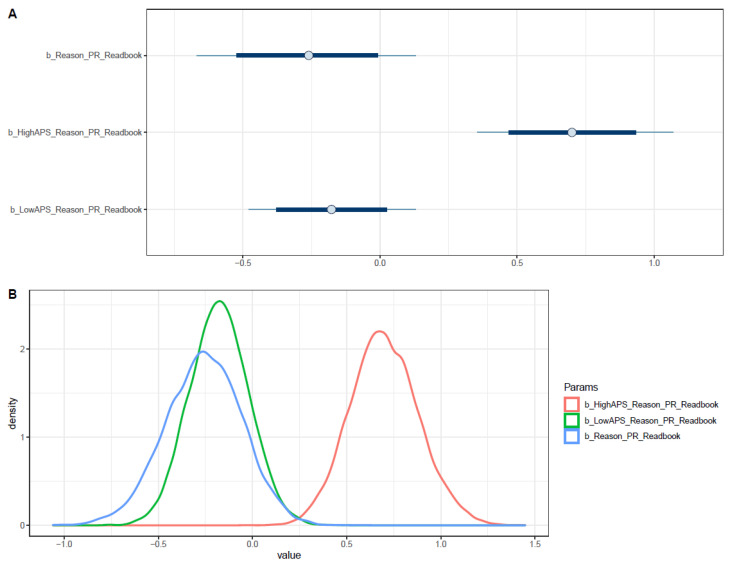
Distributions of Model 1’s posterior coefficients. (**A**)—Interval plot; (**B**)—Density plot.

The distributions of posterior coefficients are demonstrated using both interval plots and density plots in Figure 2A,B, respectively. Since the distribution of Reason_PR*LowAPS lies entirely on the positive side, and the majority of *Reason_PR*’s and Reason_PR*LowAPS’s distributions are located on the negative side, the associations’ tendencies are relatively reliable.

**Figure A6:**
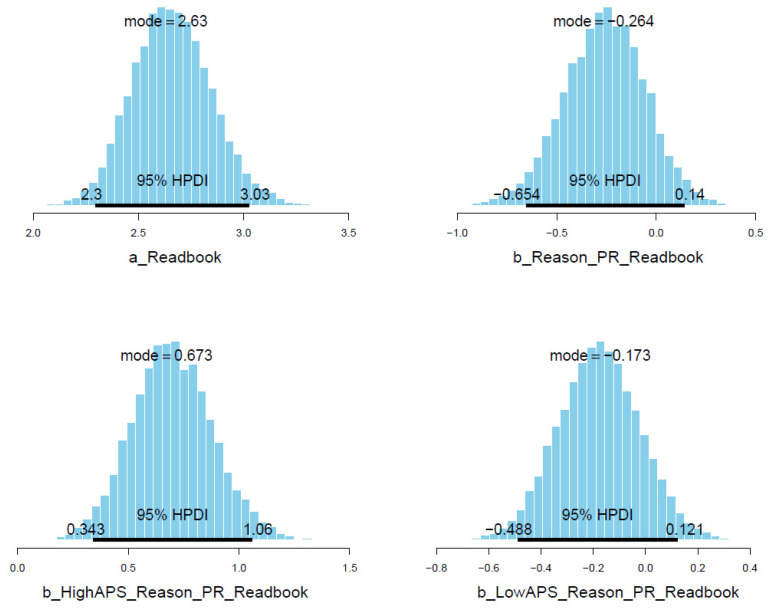
Distributions of Model 1’s posterior coefficients with HPDI at 95%.

Notably, if students with high APS choose a preferred type of book by their personal preferences, their reading interest is the most likely to be improved. Given that the Reason_PR*HighAPS’s posterior distribution at 95% Highest Posterior Distribution Intervals (HPDI) is greater than 0.34, the prior finding has high reliability and robustness (see Appendix A, Figure A6).

**Figure A7:**
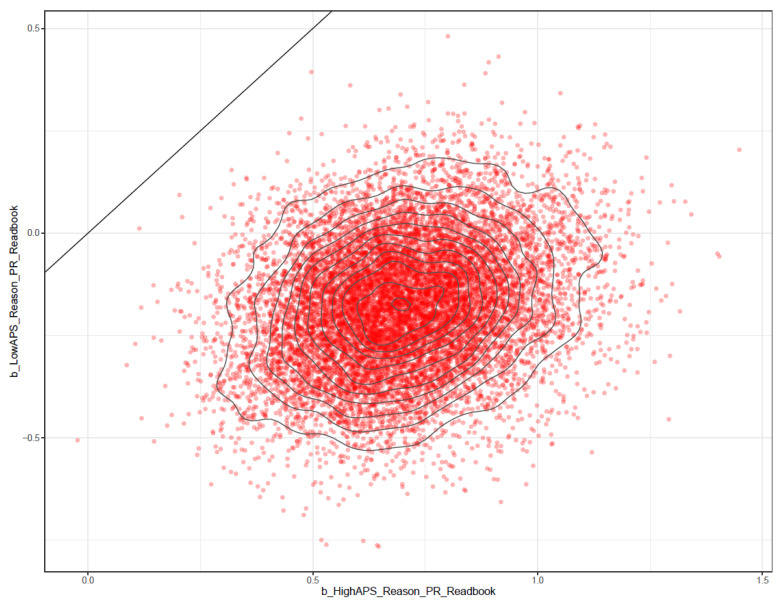
Pairwise distribution plot for model 1’s Reason_PR*HighAPS and Reason_PR*LowAPS.

The pairwise distribution between Reason_PR*HighAPS and Reason_PR*LowAPS coefficients is visualized (see Appendix A, Figure A7). While all the simulated samples are located within the positive side of Reason_PR*HighAPS (x-axis), a majority of the simulated samples lie on the negative side of Reason_PR*LowAPS (y-axis).

### 5.2. Model 2

The second model aims to check the influence of *Readstory* and its interactions with *HighAPS* and *LowAPS* on the reasons for choosing a preferred type of book. All the simulation settings are similar to those performed in Model 1 above.

**Figure A9:**
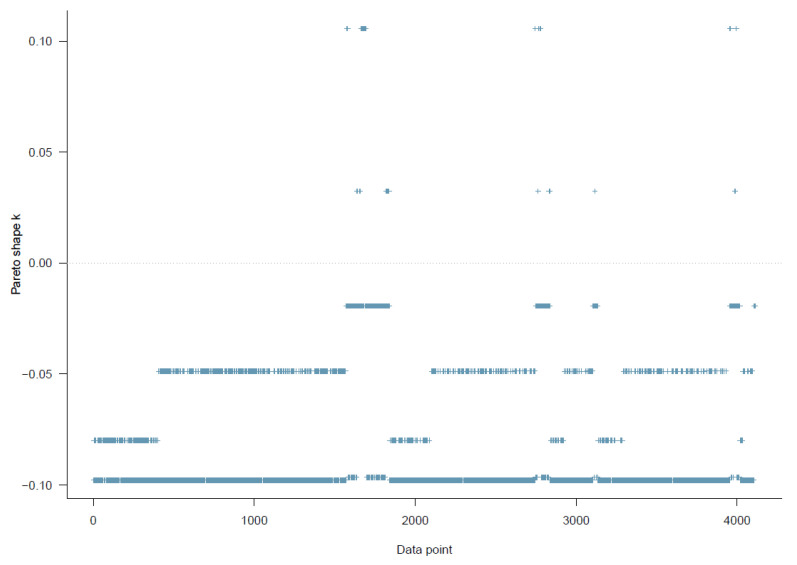
Model 2’s PSIS diagnostic plot.

All results show good statistical validity (n_eff > 1000, Rhat = 1; see Table 3) and the model exhibits a high goodness-of-fit with the data (k < 0.5; see Appendix A, Figure A9).

**Figure A10:**
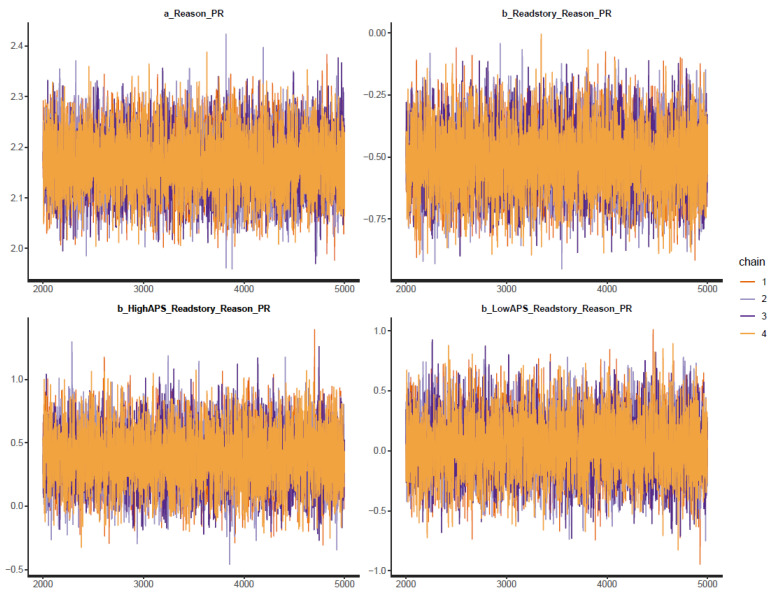
Trace plots for Model 2’s posterior coefficients.

Visually, the trace plots for Model 2’s posterior coefficients show a “clean and healthy” convergence of the Markov chains, for which all lines fluctuate around a stable central tendency and mix well with each other (see Appendix A, Figure A10).

**Figure A11:**
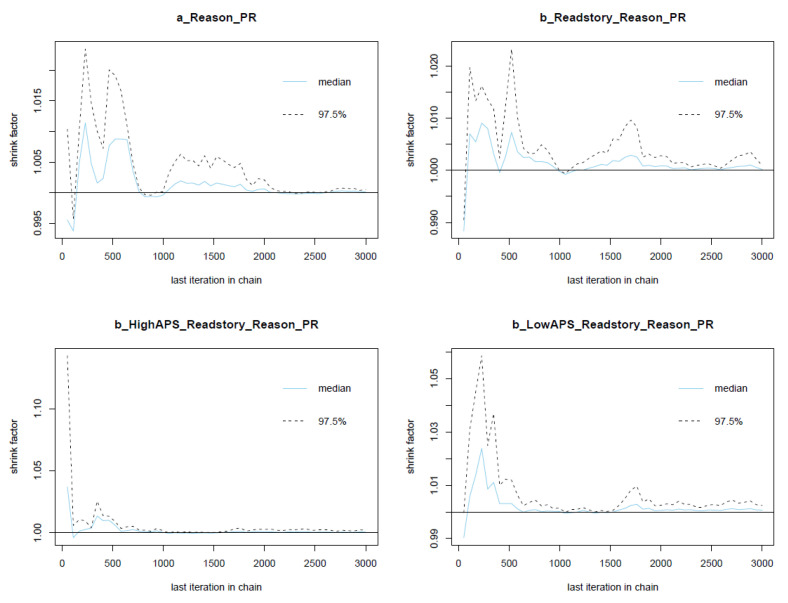
Gelman plots for Model 2’s posterior coefficients.

**Figure A12:**
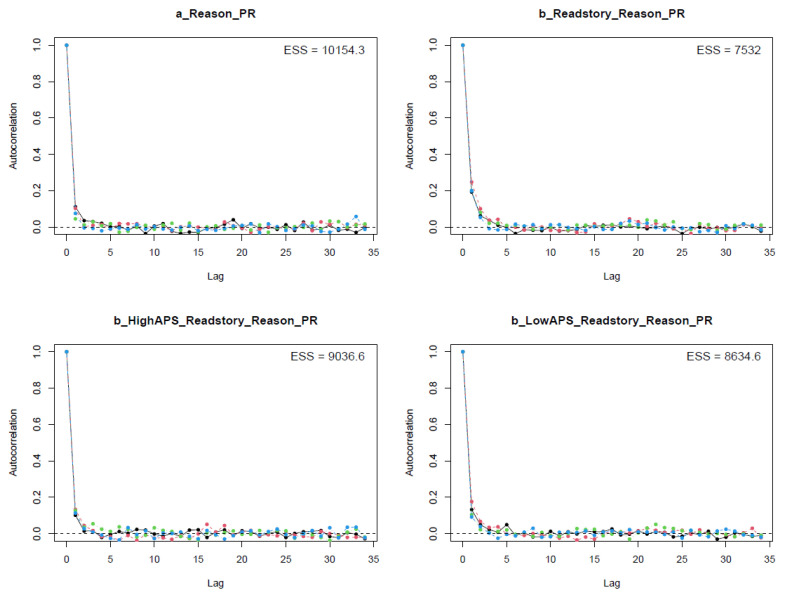
Autocorrelation plots for Model 2’s posterior coefficients.

Besides the trace plots, the Gelman plots also demonstrate a good signal of convergence in which the shrink factor drops rapidly to one during the warm-up process (see Appendix A, Figure A11). The Markov chain central limit theorem holds in Model 2’s simulation as the autocorrelations among posterior samples reduce to zero swiftly (see Appendix A, Figure A12).

**Figure 3:**
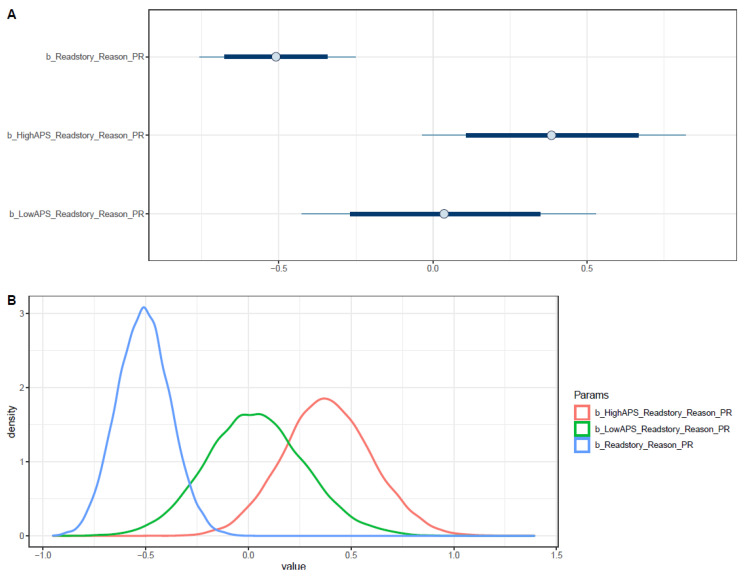
Distributions of Model 2’s posterior coefficients. (**A**)—Interval plot; (**B**)—Density plot.

**Figure A13:**
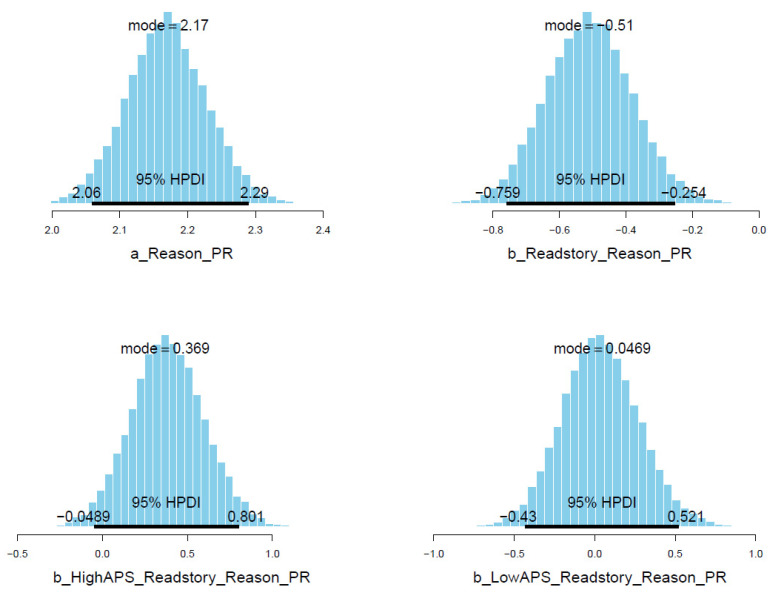
Distributions of Model 2’s posterior coefficients with HPDI at 95%.

Parental book reading was found to have a negative association with personal preference as the reason to select a preferred type of book ($\mu_{Readstory}$ = −0.51 and $\sigma_{Readstory}$ = 0.13), which also means parental book reading is positively associated with the reason being recommendations. However, when adding interaction with high APS, the influence of parental book reading on children’s reason to select a preferred type of book was moderated ($\mu_{Readstory*HighAPS}$ = 0.39 and $\sigma_{Readstory*HighAPS}$ = 0.21). The interaction between parental book reading and students’ low APS did not yield a meaningful effect as its posterior distributes around zero. Figure 3 indicates that the distribution of *Readstory* lies wholly on the negative side, while that of Readstory*HighAPS is mostly situated on the positive side. These distributions suggest a strong negative association between parental story reading and the reason to select a preferred type of book being preferences and the reliable moderating effect of high APS. The distributions of *Readstory* and Readstory*LowAPS with HPDI at 95% confirm their associations’ robustness with *Reason_PR* (see Appendix A, Figure A13).

**Figure A14:**
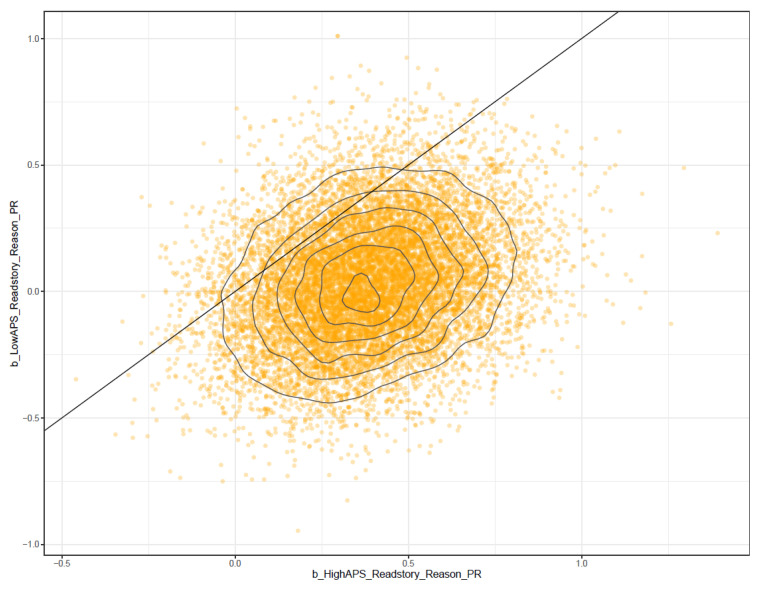
Pairwise distribution plot for model 2’s Readstory*HighAPS and Readstory*LowAPS.

The pairwise distribution between Readstory*HighAPS (y-axis) and Readstor*LowAPS (y-axis) indicates a clear moderating effect of high APS on the impact of parental story reading on the reason to select a preferred type of book was visualized (see Appendix A, Figure A14).

### 5.3. Model 3

Model 3 attempts to examine the effects of parental book reading and its interactions with students having high or low APS on book reading interest. Bayesian MCMC simulation settings are similar to those performed with the other two models (5000 iterations, 2000 warm-up iterations, four Markov chains, and ‘uninformative’ priors).

**Figure A16:**
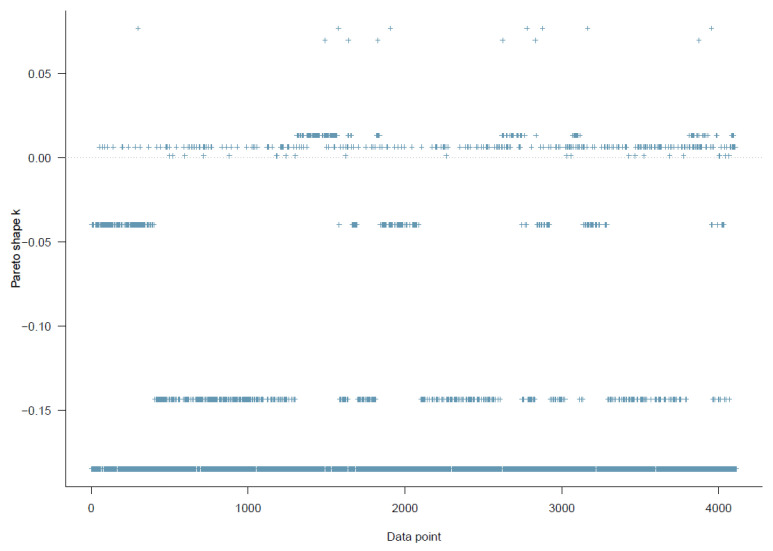
Model 3’s PSIS diagnostic plot.

All results show good statistical validity for the model’s convergence: n_eff > 1000 and Rhat = 1 (see Table 4), while the PSIS diagnostic plot implies a high goodness-of-fit between Model 3 and the data (see Appendix A, Figure A16).

**Figure A17:**
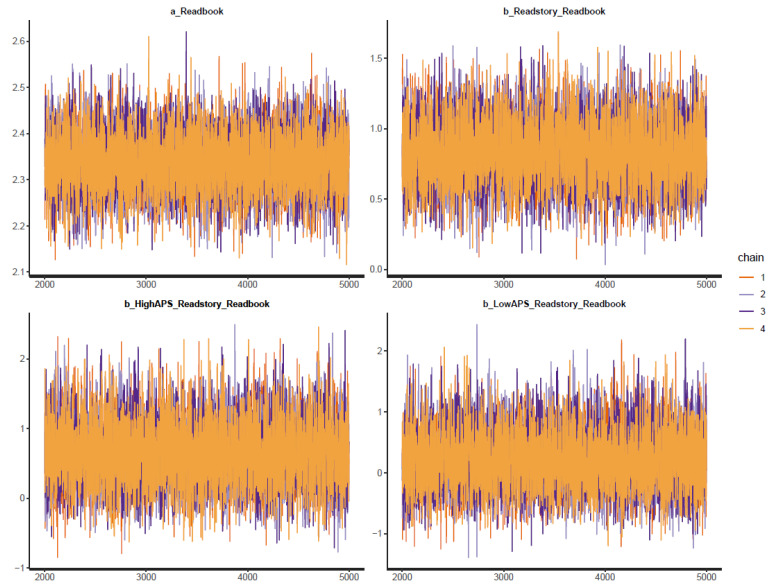
Trace plots for Model 3’s posterior coefficients.

**Figure A18:**
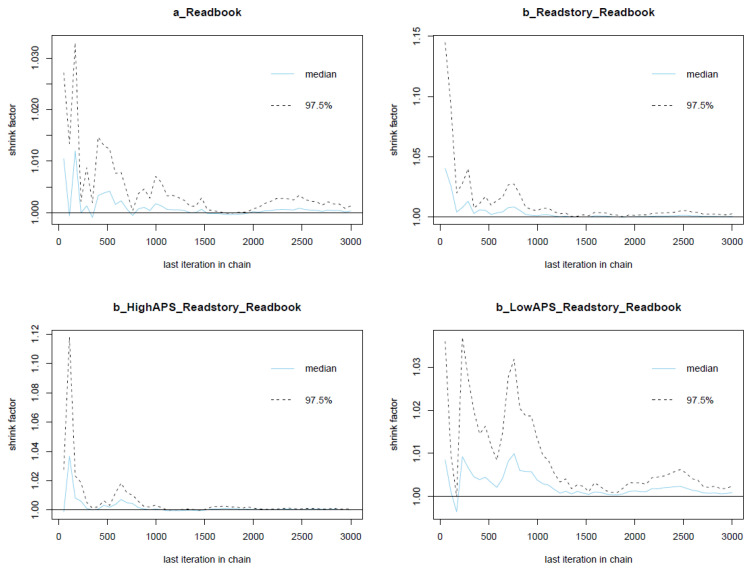
Gelman plots for Model 3’s posterior coefficients.

**Figure A19:**
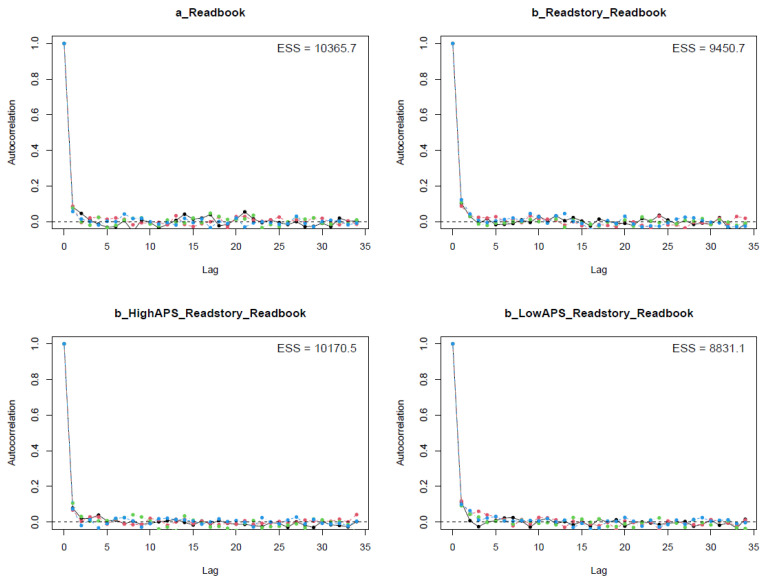
Autocorrelation plots for Model 3’s posterior coefficients.

The trace plots, Gelman plots, and autocorrelation plots indicate a good sign of convergence, and the Markov property is satisfied (see Appendix A, Figure A17, Figure A18 and Figure A19, respectively).

**Figure 4:**
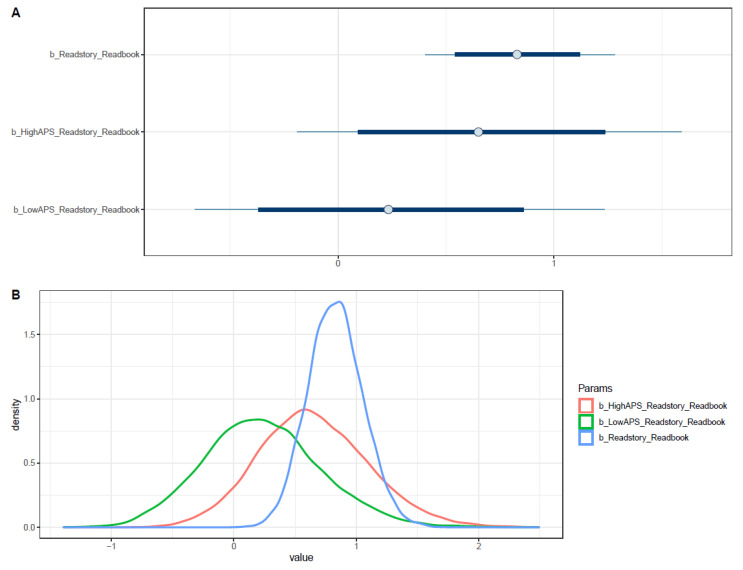
Distributions of Model 3’s posterior coefficients. (**A**)—Interval plot; (**B**)—Density plot.

Parental book reading was found to have a positive association with students’ reading interest ($\mu_{Readstory}$ = 0.83 and $\sigma_{Readstory}$ = 0.22). Both parental book reading’s interactions with high and low APS resulted in the same direction of influence ($\mu_{Readstory*HighAPS}$ = 0.65; $\sigma_{Readstory*HighAPS}$ = 0.44; and $\mu_{Readstory*LowAPS}$ = 0.23; $\sigma_{Readstory*LowAPS}$ = 0.48) with high APS yielded clearer and greater impact. All coefficients’ distributions lie almost entirely on the positive side, except for Readstory*LowAPS (see Figure 4).

**Figure A20:**
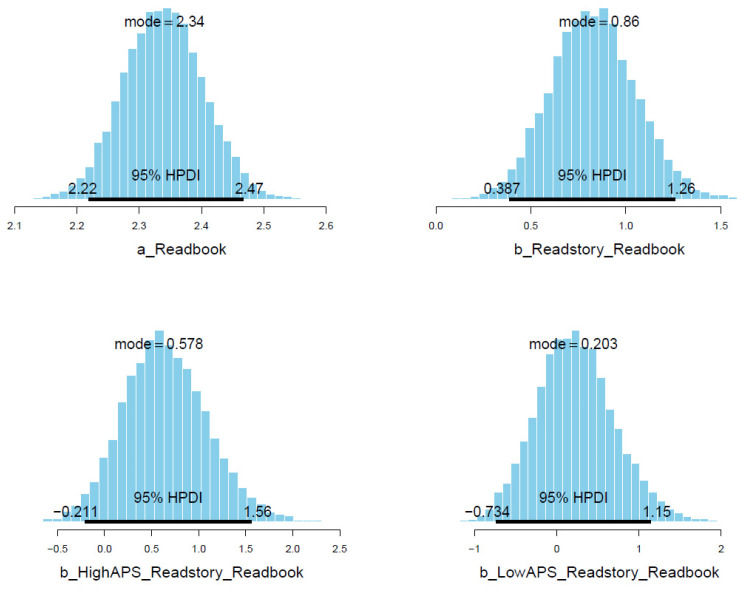
Distributions of Model 3’s posterior coefficients with HPDI at 95%.

**Figure A21:**
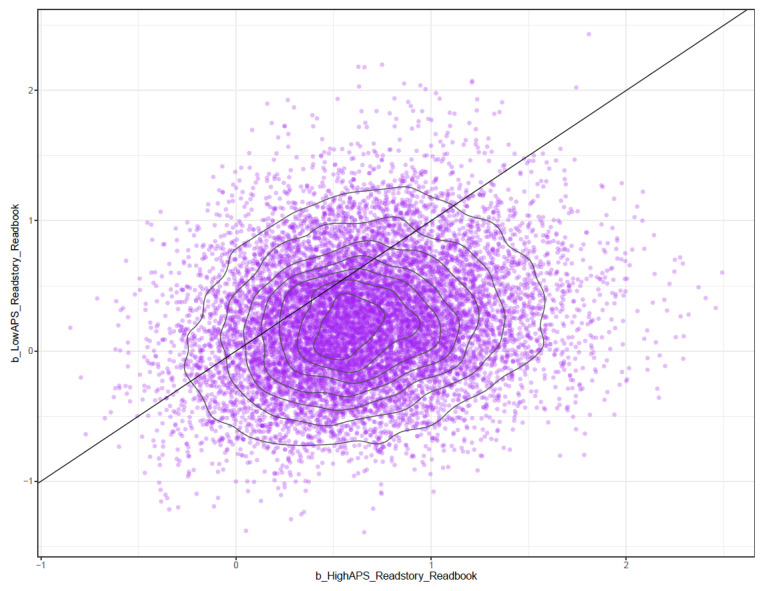
Pairwise distribution plot for model 3’s Readstory*HighAPS and Readstory*LowAPS.

Even though Readstory*LowAPS had a positive influence on book reading interest among children, its effect is contentious (see Appendix A, Figure A20 and Figure A21).

## 6. Discussion

The present study explores the psychological mechanism of secondary students’ book reading interest and how home intellectual culture can contribute to that process. Applying the Mindsponge framework, we identified two fundamental approaches that can help raise students’ book-reading interest: recommendation and self-determination. Other questions also arise: Is the book reading interest in students with high and low academic achievements similarly affected through a similar pathway? How does home scholarly culture help raise book-reading interest? These research questions were investigated using Bayesian analysis aided by MCMC simulation on a dataset of approximately 5000 Vietnamese secondary students.

Foremost, we want to address this study’s limitations, as transparency is beneficial to scientific investigation [62]. First, the data was collected in Northern Vietnam only. Second, parental book reading is just a part of the home scholarly culture. Third, regarding the book recommendation factor, due to its general question in the survey, the manner of recommendation (positive or controlling) is unknown. Forth, although all public and most domestic private secondary schools in Vietnam employ standardized educational structures (e.g., same textbooks, classroom arrangements, and examination systems) issued by the Minister of Education, schools in poor rural areas or schools for gifted students may not strictly follow the standards (and some international schools follow their own programs). However, we believe that our approach of examining book reading interest in terms of information processing using the Mindsponge psychological mechanism can be universally applied to various situations, not only in Vietnam but also in other world regions.

### 6.1. Book Recommendation or Personal Preference?

Our result from the first model showed that students who choose a preferred type of book by recommendation—instead of personal preference—are more likely to be interested in reading books. However, being a student with high APS strongly negates this effect. In other words, high-APS students are more interested in reading books when they can follow their personal preferences. This result indicates the different thought processes when choosing a preferred type of book between students with high performance in STEM subjects and those with low performance. Step-by-step reasoning using the Mindsponge framework is presented as follows. Moreover, the visualization of information-processing mechanisms of low- and high-achieving students is illustrated in Figure 1.

Students who like to read books must perceive the value of reading a book as more beneficial than its cost. This value includes both the information and the information source (information needs to pass through trust-evaluation filters). Students who choose the book by recommendations from trusted people (teachers, friends, and parents) consider added value from these external sources. When recommending books to children, the one who recommends should have credibility, such as being knowledgeable or a role model of book reading [63]. This trust increases the perceived value of the source of information—in this case, the one who recommend. On the other hand, those who choose books based on their preferences consider added value from their internal source (self). Early research recorded that children feel engaged and express knowledge to peers and educators when choosing their books [64].

Deducting from the corresponding behaviors, students who prefer recommendation should have a higher relative perceived benefit of external sources than internal sources. In contrast, students who prefer autonomy will not like the book given by an external decision (recommendation) because they perceive the internal information source’s added value to be higher than the external source. In brief, autonomous students will be more unlikely to read books being recommended to them.

Students with high performance in STEM subjects tend to have higher perceived self-efficacy [65,66], which might make them more autonomous [67,68]. While APS in STEM subjects does not represent a student’s overall academic performance, the related social environment can heavily affect their self-perception [69,70]. This is particularly severe in developing Asian countries like Vietnam, where the education system and parents perceive STEM score to be the most (if not only) significant predictor of future success. Such social stigma causes students to base their self-image on this context as well. Peer group notions (“good” and “bad” students) may further reinforce the notion. A study on Vietnamese secondary school students showed that if students perceive math to be difficult and the classroom environment competitive, their self-esteem will be negatively affected [71].

With high perceived self-efficacy, high-APS students are more confident that their decisions hold more benefits for themselves. This sense of autonomy leads to the difference in the thought process of high-APS students compared to those with low-APS. Model 1 showed how it affects the cost-benefit judgment regarding the information source, in this case, recommendation or personal preference. Inductively, this result supports the presented information processing model based on the Mindsponge theory regarding the perception of information’s value. There is a difference in such perception between high and low-achieving students. We will continue the discussions on this aspect in the following sections and suggest practical educational approaches accordingly.

### 6.2. Influence of Parental Book Reading on Source Preference

Given the importance of home scholarly culture and parents–children interaction toward students’ academic performance [12,72], we examine the mechanism of how the act of parents reading books to children influences children’s reading behaviors. Based on the first model results, logically, we expect that the influence on the added value of the information source will affect the corresponding perception toward it. Here in the second model, we tested if support increasing perceived value of external source would have the projected effect of overall increasing book recommendation preference but less impactful in high-APS students. As expected, results show that students with parental book reading tend to choose the preferred book type by recommendation. Consistent with the mechanism discussed above, we see that being a high-APS student negates this effect.

Using the same pathways, we briefly explain the underlying thought process here. Reading a book for a child over time will increase the acceptance rate of information passing through the trust-evaluation filter regarding the information source being parents; thus, it increases the value of the external source (recommendation), leading to a higher rate of acceptance from this source. It is worth noting that the increase of trust toward parents results from complex interactions during the reading sessions and not simply from the act of reading the text aloud [73]. Students with higher autonomy (as discussed, are those with higher APS) already hold a higher perceived value of the internal source (self’s decisions) as opposed to the external one (recommendation). Thus, it is harder to shift the cost-benefit scale between recommendation and personal preference in the direction of recommendation. However, interactions between children and their parents are not straightforward, but rather complex processes of exchanging information and adapting to such interactions. We explore this aspect further through the discussion on the result of Model 3 below.

### 6.3. How Parental Book Reading Influences Reading Interest

The third model results showed that students whose parents read books to are more likely to be interested in reading books. This is a natural strategy for encouraging children to read books and develop overall curiosity [63]. Even at the young age of learning how to read, children are naturally attracted by stories read to them and will attempt reading books by themselves [74,75].

Notably, high-APS students showed the same pattern, but they also have a stronger effect than the low-APS group. At first glance, this result may appear inconsistent with the above mechanism where higher autonomy leads to the negation of recommendation—which was increased by parental book reading. However, it is important to notice that the results do not indicate the direction of influence between children and parents. While parents’ act of reading books can lead to children liking those books (which were chosen by parents), the other direction is also equally possible: children choose their favorite books and have their parents read to them. It was found that children feel intrinsically rewarded toward their favorite books and require others to read to them from a very young age [74].

The unidirectionality in the result of Model 3 suggests that while parental book reading increases reading interest in both high and low-APS groups, each type’s pathway is different. Low-APS students who prefer recommendations will have their parents choose and read books for them, reinforcing the preference for external sources (as shown in Model 2), making them like reading books more (as shown in Model 1). In contrast, high-APS students who tend to prefer autonomous decision-making will choose their favorite books and have their parents read to them, further improving book enjoyment (relatively more likely to read books as shown in Model 3), while still maintaining their favor of personal preference over recommendation (as shown in Model 1 and 2). Regarding the results from Models 2 and 3, we can see that the act of parents reading a book to children is quite complex in terms of information receiving, evaluating (accept or reject), and generating (react). Here, we speculate that when the parents know their children’s personal preferences, they will tend to support the children in the same direction as we currently deem as the most likely possibility.

### 6.4. Implications for Educational Policy

Based on the reading preference mechanism presented above, we advocate the following focus points for educational policymaking, not only in Vietnam but also on a global level.

#### 6.4.1. Recommendation Is Important for Students with Low Self-Efficacy

Students who are uncertain about choosing good information sources need help in the form of specific recommendations from parents and teachers (note that recommendation is different from the negative form of “control”). This improves their knowledge and interest in the subjects and helps build stronger trust between students and educators, making it easier to help them later. While choosing books for children, we should be careful and consider many different aspects as there may be unexpected negative influences that adults are normally not aware of [76]. With the advantages of modern technology, personalized recommendation systems for children on online platforms are also possible and proven efficient [77].

Students with low self-efficacy receive more help from educators [78]. However, as the students improve their academic ability, it is important to give them higher autonomy in decision-making. Socio-cultural factors are also very important to self-efficacy. Research showed that Asian students have relatively low math self-efficacy and high math anxiety despite high math performance than a more balanced perception in Western European students [79].

#### 6.4.2. Autonomy Is Important for Students with High Self-Efficacy

High-achieving students put a high value on their own decisions. It is counter-effective to go against the sense of autonomy in attempts to support students with this type of mentality. On the contrary, we should provide support in alignment with the students’ interests and vocations, such as through autonomy support programs in secondary school’s classroom environment [80]. This is particularly crucial for outstanding groups like those in schools for gifted students–as non-identified high-achieving and identified gifted students share the same psychosocial self-perceptions [81]. Specific and detailed instructions may be more beneficial to other groups. Still, high-achieving students highly value their own ways of studying, which is a key factor when creating special programs (e.g., textbooks for gifted students) and designing educational approaches (e.g., teacher–student interaction).

#### 6.4.3. Student–Educator Relationship Should Be Considered in Both Directions

Traditionally, education is often seen as one-sided, especially in conservative Asian cultures. A proactive attitude is required to support the young generations of students and improve the whole national system and perspective about science [82]. Our study has reinforced the importance of utilizing appropriate approaches for students with different thought processes in improving academic engagement. As the students learn from the educators how to learn more efficiently, they also teach the educators how to teach them more effectively [83]. A good children–educators relationship may include the opposite side of recommendation: educators read books that are recommended by the children [63]. This can help foster a deeper connection and understanding of the child’s interests and perspectives. Building a sustainable student-educator relationship requires identifying proper needs and interventions and developing mutual trust and respect [84,85]. In terms of information processing, it is a continuous and dynamic loop of receiving, evaluating, and generating between both actors (students and educators). Additionally, we can see that personalized education can become more effective when students’ preferences are used instead of their performance (in terms of score) for identifying suitable support.

## Figures and Tables

**Table 1:** Variable description.

Name	Variable	Data Type	Description
Reading Interest	*Readbook*	Binary	Whether the student likes reading books or not. “Yes” is coded as 1 and “No” as 0.
Students with high APS	*HighAPS*	Binary	Students with an average score of STEM-related subjects “APS45” ≥ 8, coded as 1, others are coded as 0.
Students with low APS	*LowAPS*	Binary	Students with an average score of STEM-related subjects “APS45” < 5, coded as 1, others are coded as 0.
Reason for choosing a preferred type of book	*Reason_PR*	Binary	Whether the reason is a recommendation or personal preference. “Personal preference” is coded as 1; “Recommendation” is coded as 0.
Parental book reading	*Readstory*	Binary	Whether a student’s parents read stories for him/her. “Yes” is coded as 1 and “No’ as 0.

**Table 2:** Model 1’s simulated posterior coefficients.

Parameters	Mean	SD	n_eff	Rhat
*Constant*	2.66	0.19	5986	1
*Reason_PR*	−0.26	0.20	5853	1
Reason_PR*HighAPS	0.70	0.18	8140	1
Reason_PR*LowAPS	−0.18	0.16	8140	1

**Table 3:** Model 2’s simulated posterior coefficients.

Parameters	Mean	SD	n_eff	Rhat
*Constant*	2.17	0.06	9511	1
*Readstory*	−0.51	0.13	7583	1
Readstory*HighAPS	0.39	0.21	8664	1
Readstory*LowAPS	0.04	0.24	9172	1

**Table 4:** Model 3’s simulated posterior coefficients.

Parameters	Mean	SD	n_eff	Rhat
*Constant*	2.34	0.06	10,555	1
*Readstory*	0.83	0.22	9247	1
Readstory*HighAPS	0.65	0.44	9856	1
Readstory*LowAPS	0.23	0.48	8741	1

**Figure A1:**
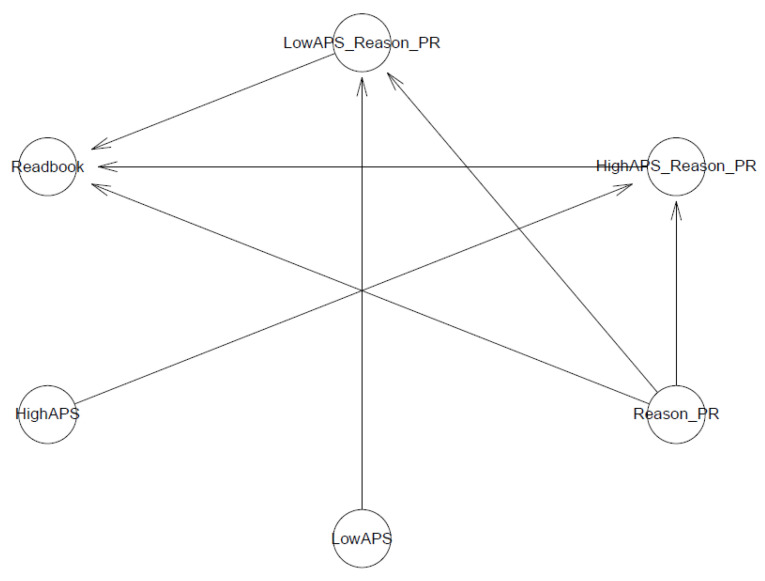
Model 1’s logical network.

**Figure A8:**
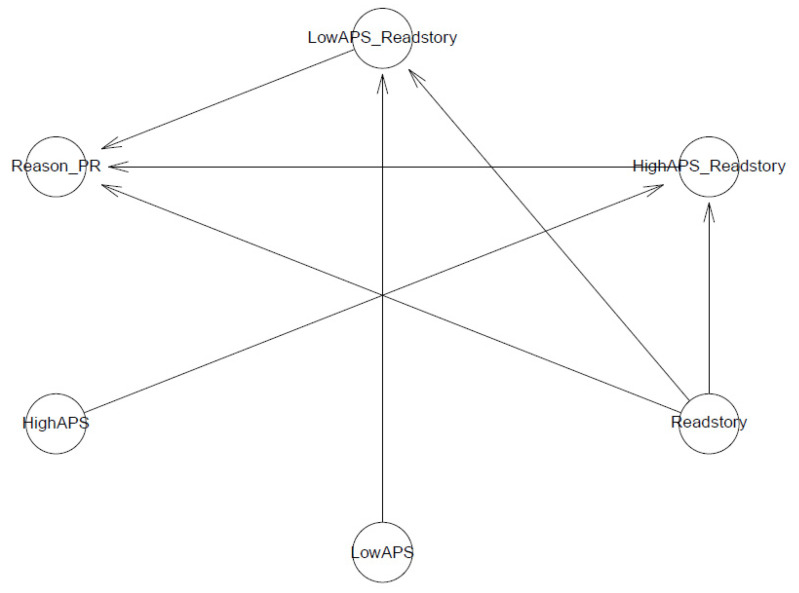
Model 2’s logical network.

**Figure A15:**
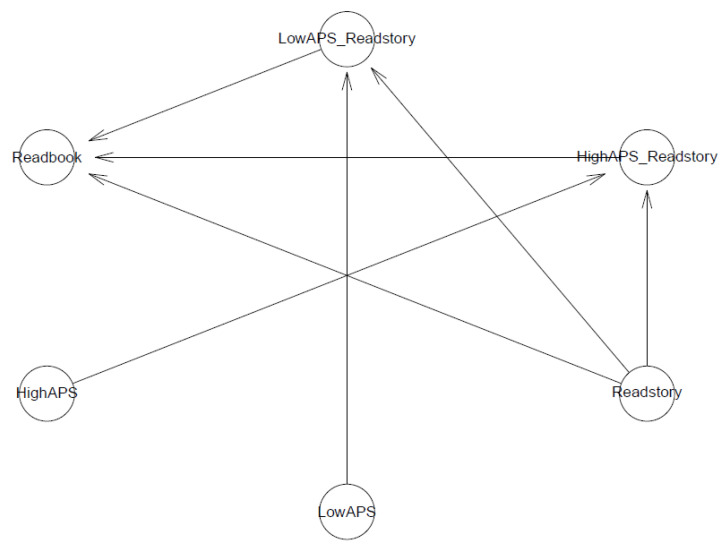
Model 3’s logical network.

## Data Availability

The dataset description open access article is available at DOI:10.1162/dint_a_00091.

## Supplementary Materials

Data, codes, and additional analysis figures were uploaded onto the OSF server, DOI:10.17605/OSF.IO/UCZDW.
