# Supplementary material for: Home Scholarly Culture, Book Selection Reason, and Academic Performance: Pathways to Book Reading Interest among Secondary School Students
Source: Eur J Investig Health Psychol Educ. 2021 Jun 1;11(2):468–95. doi: 10.3390/ejihpe11020034 (PMC8314380; doi:10.3390/ejihpe11020034)
Supplement: Supplementary file 1 [file ejihpe-11-00034-s001.zip › Analysis validity figures.pdf]

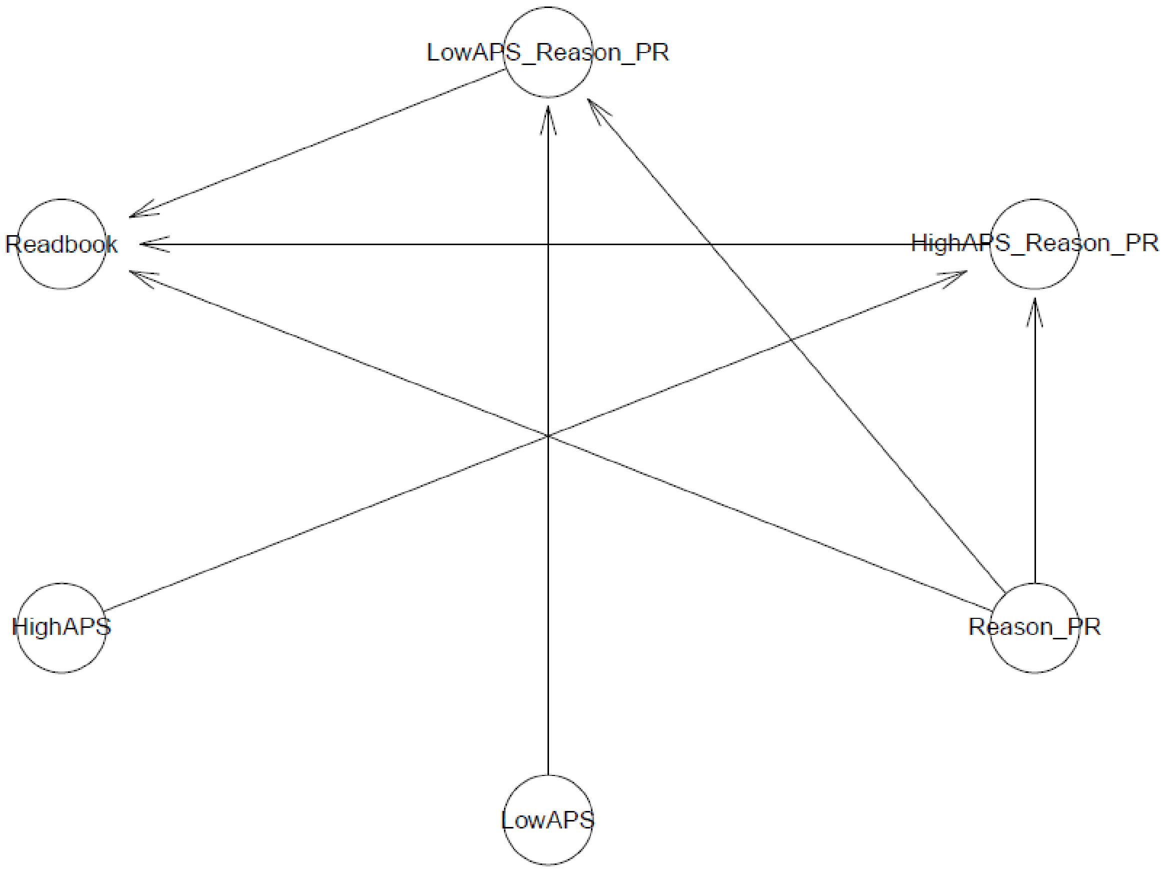

Figure S1: Model 1's logical network

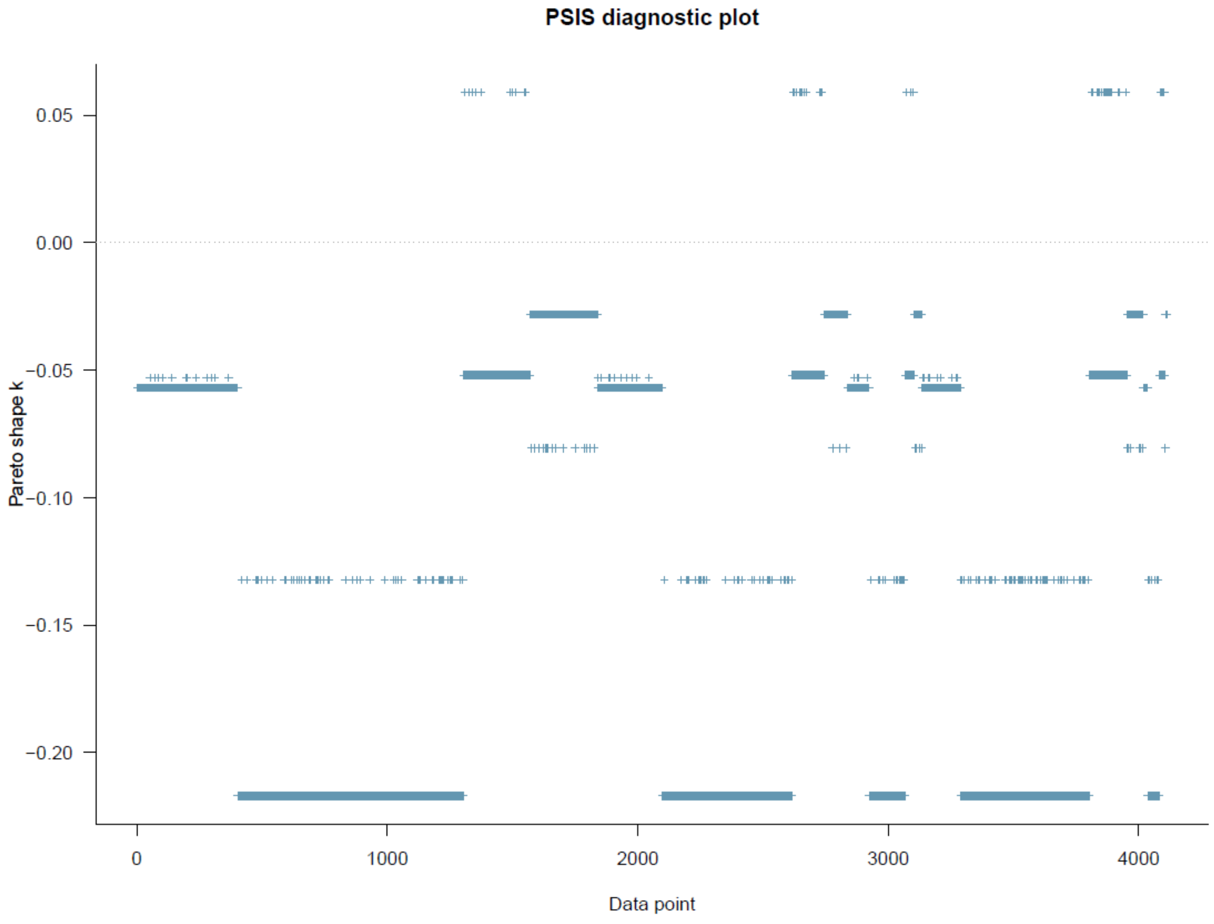

Figure S2: Model 1's PSIS diagnostic plot

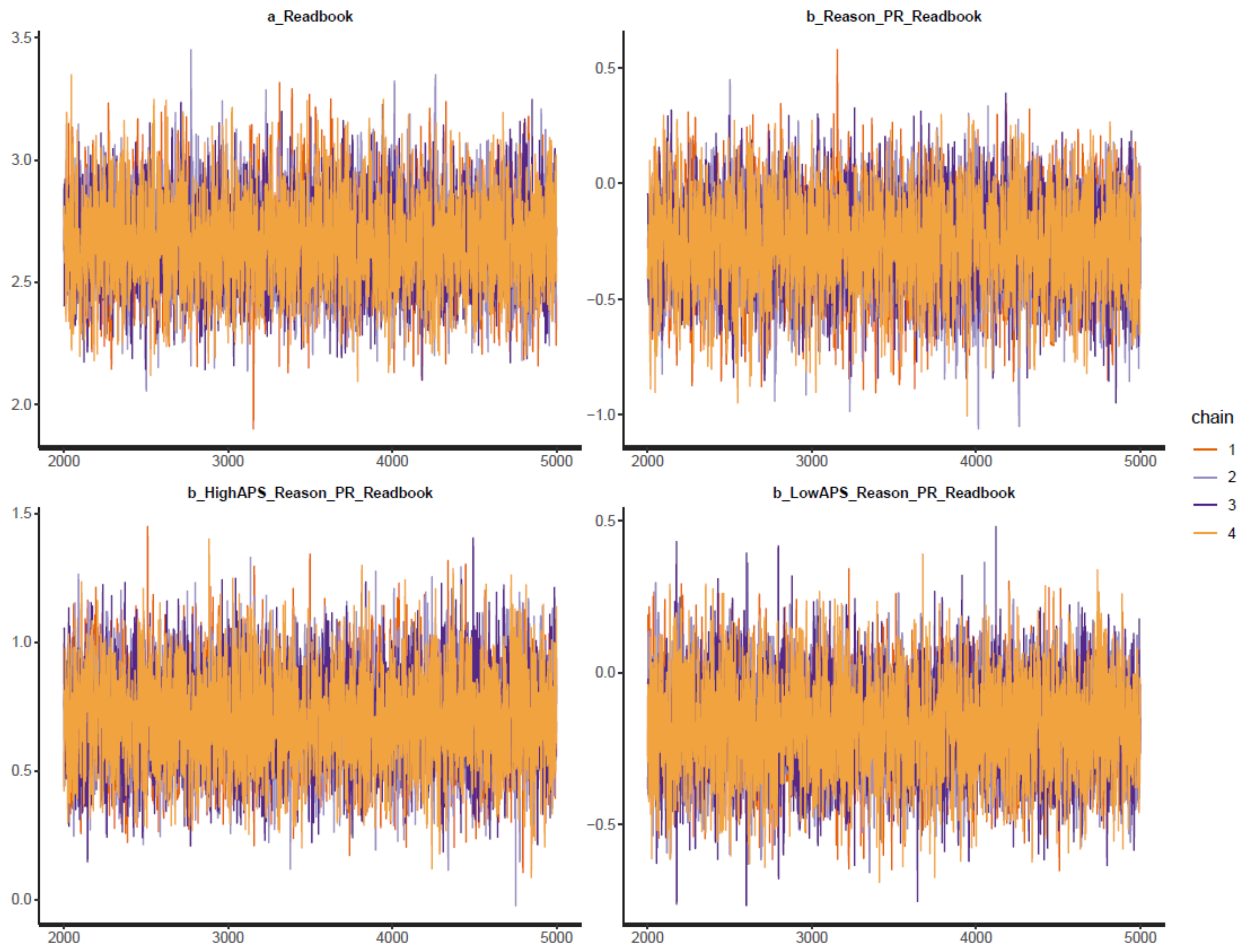

Figure S3: Trace plots for Model 1's posterior coefficients

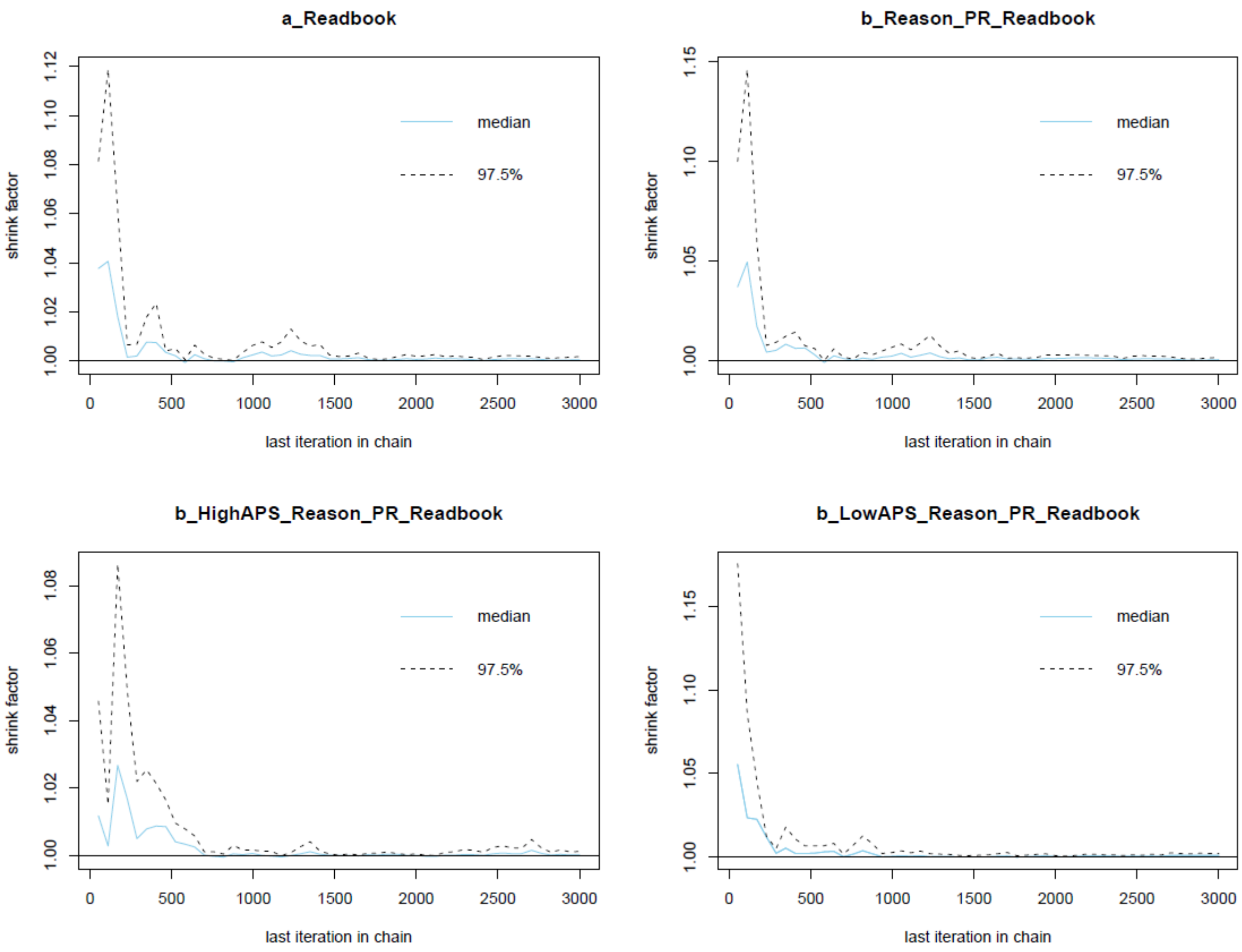

Figure S4: Gelman plots for Model 1’s posterior coefficients

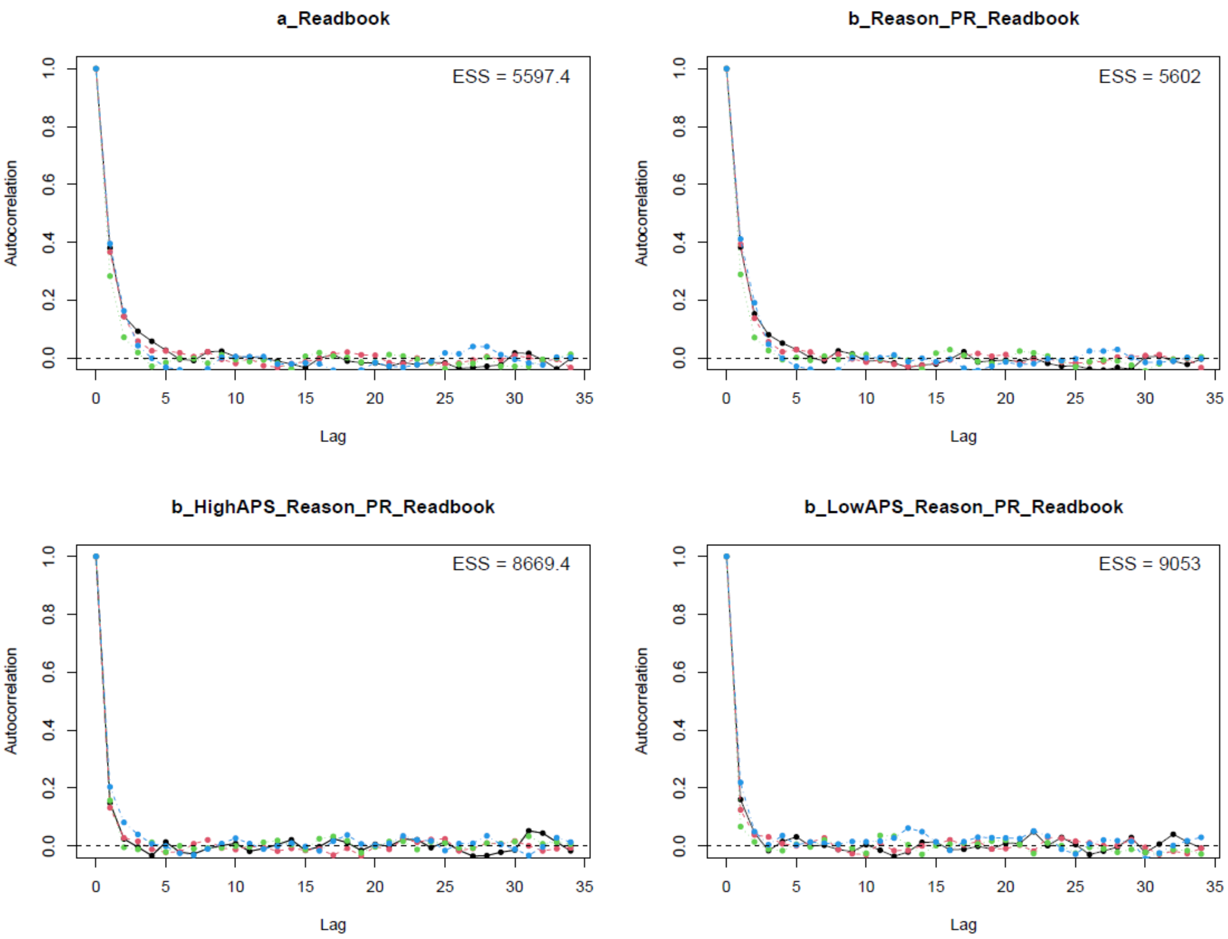

Figure S5: Autocorrelation plots for Model 1’s posterior coefficients

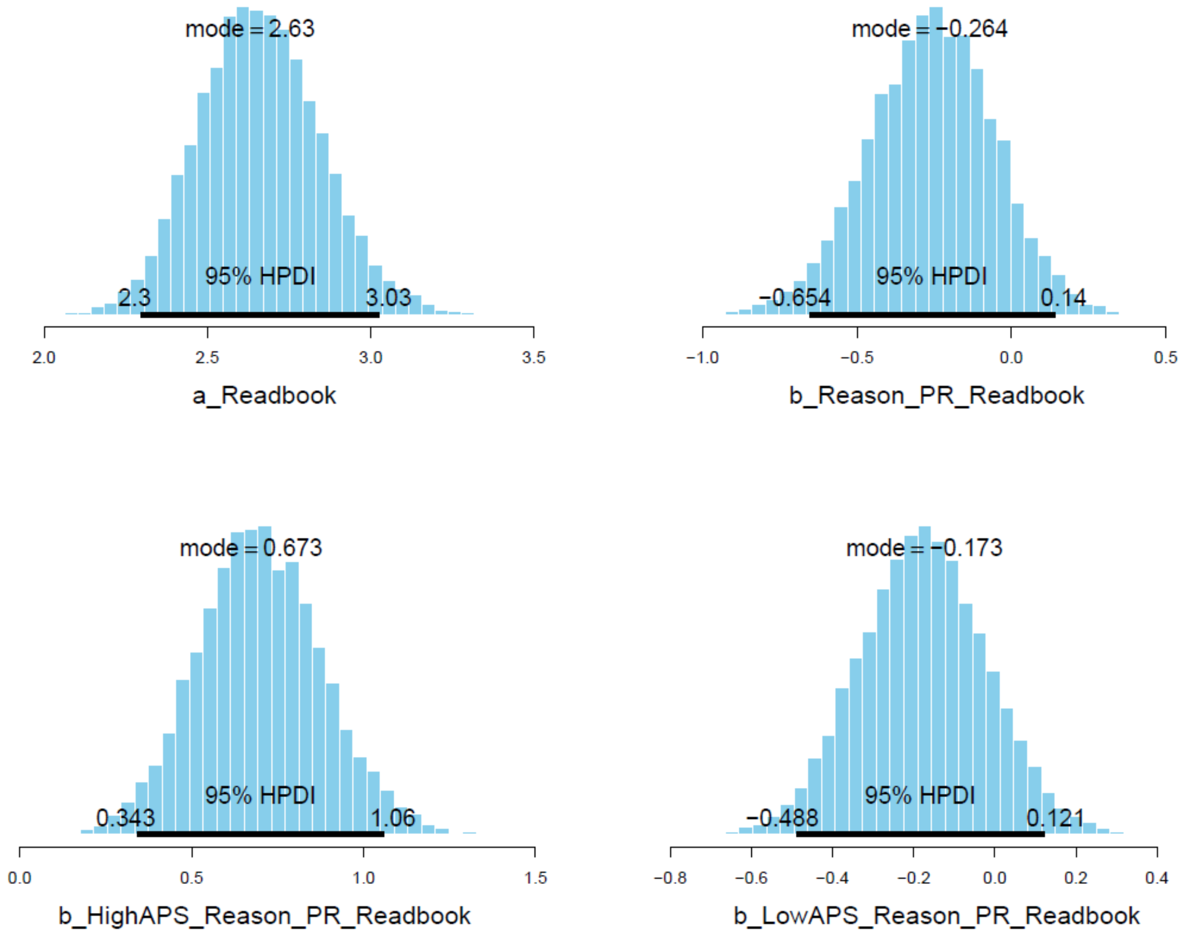

Figure S6: Distributions of Model 1’s posterior coefficients with HPDI at 95%

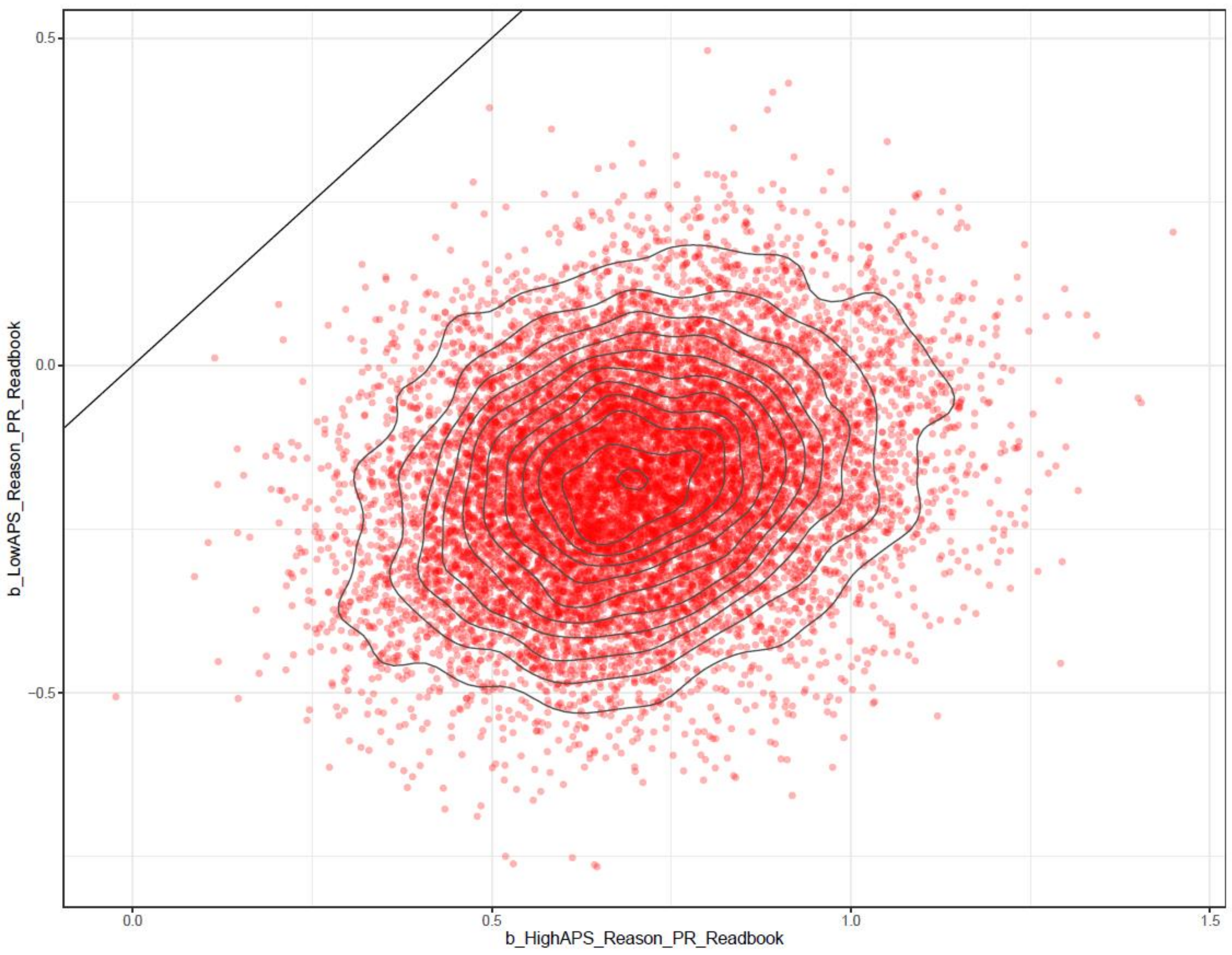

Figure S7: Pairwise distribution plot for model 1's *Reason_PR*HighAPS* and *Reason_PR*LowAPS*

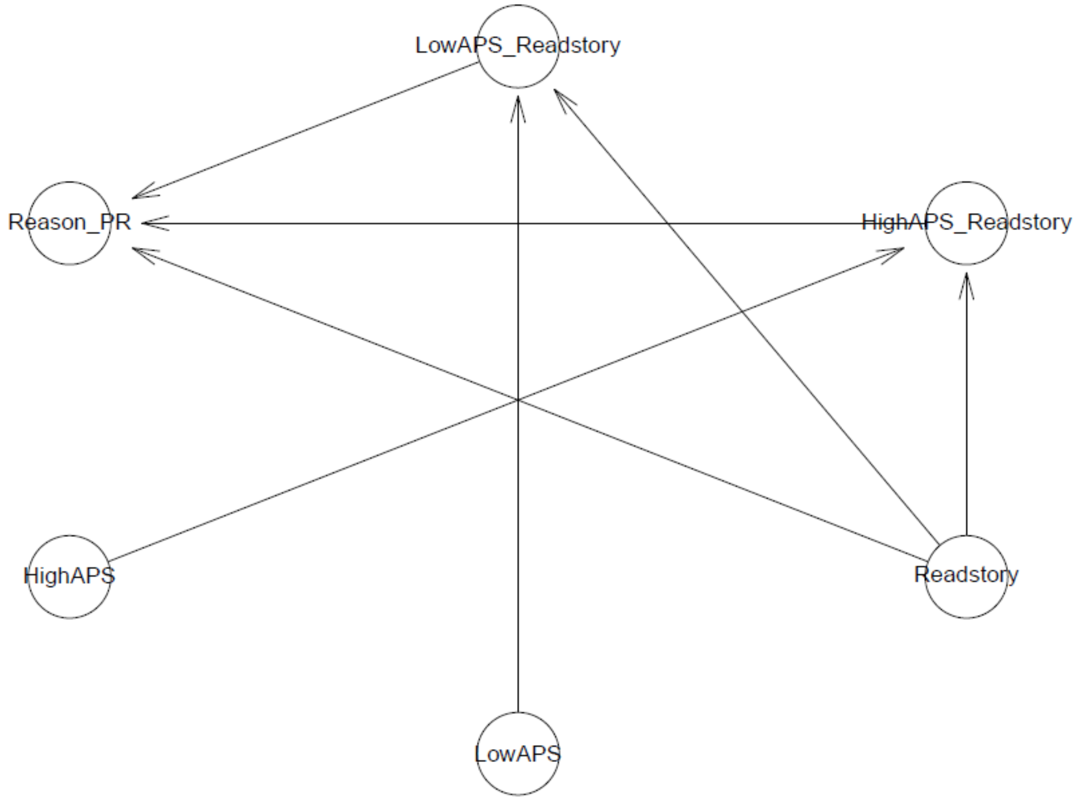

Figure S8: Model 2's logical network

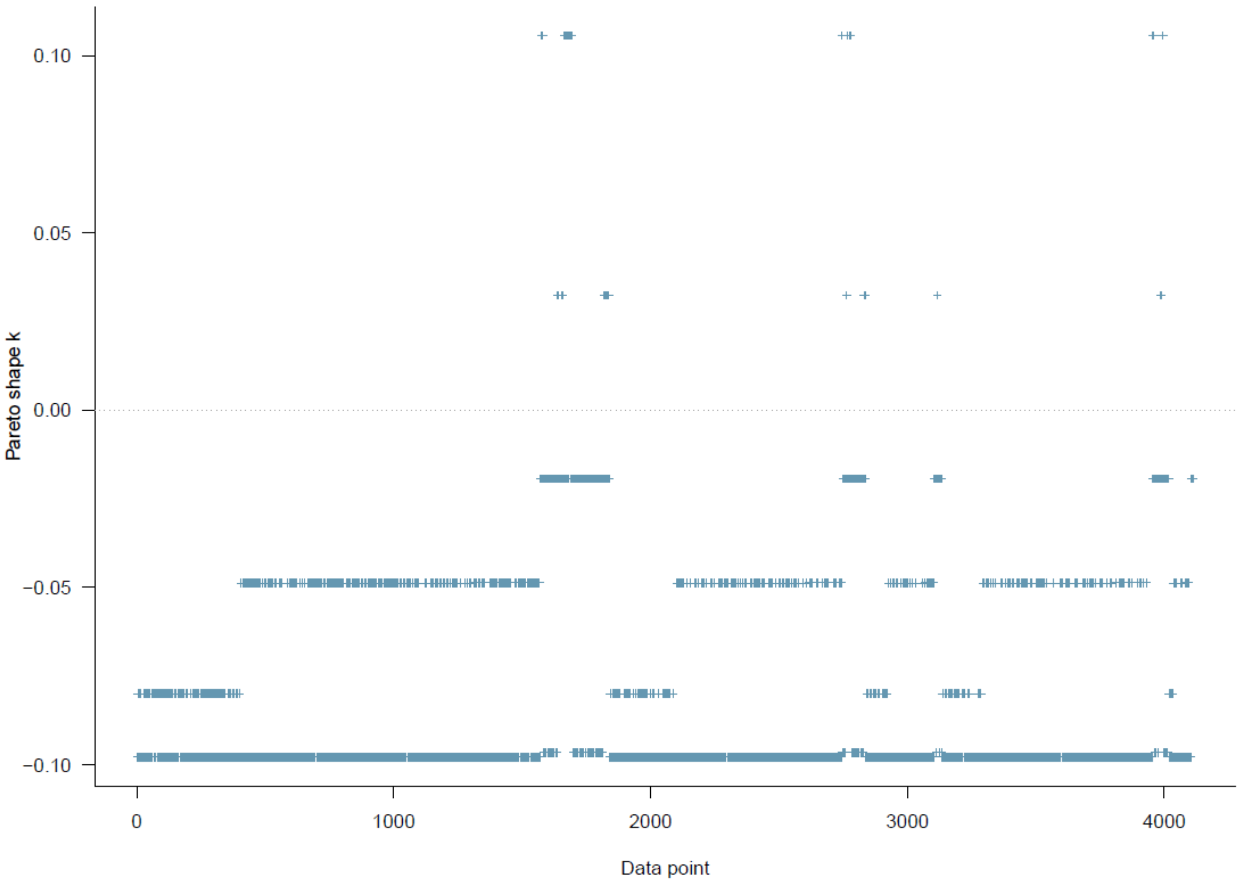

Figure S9: Model 2's PSIS diagnostic plot

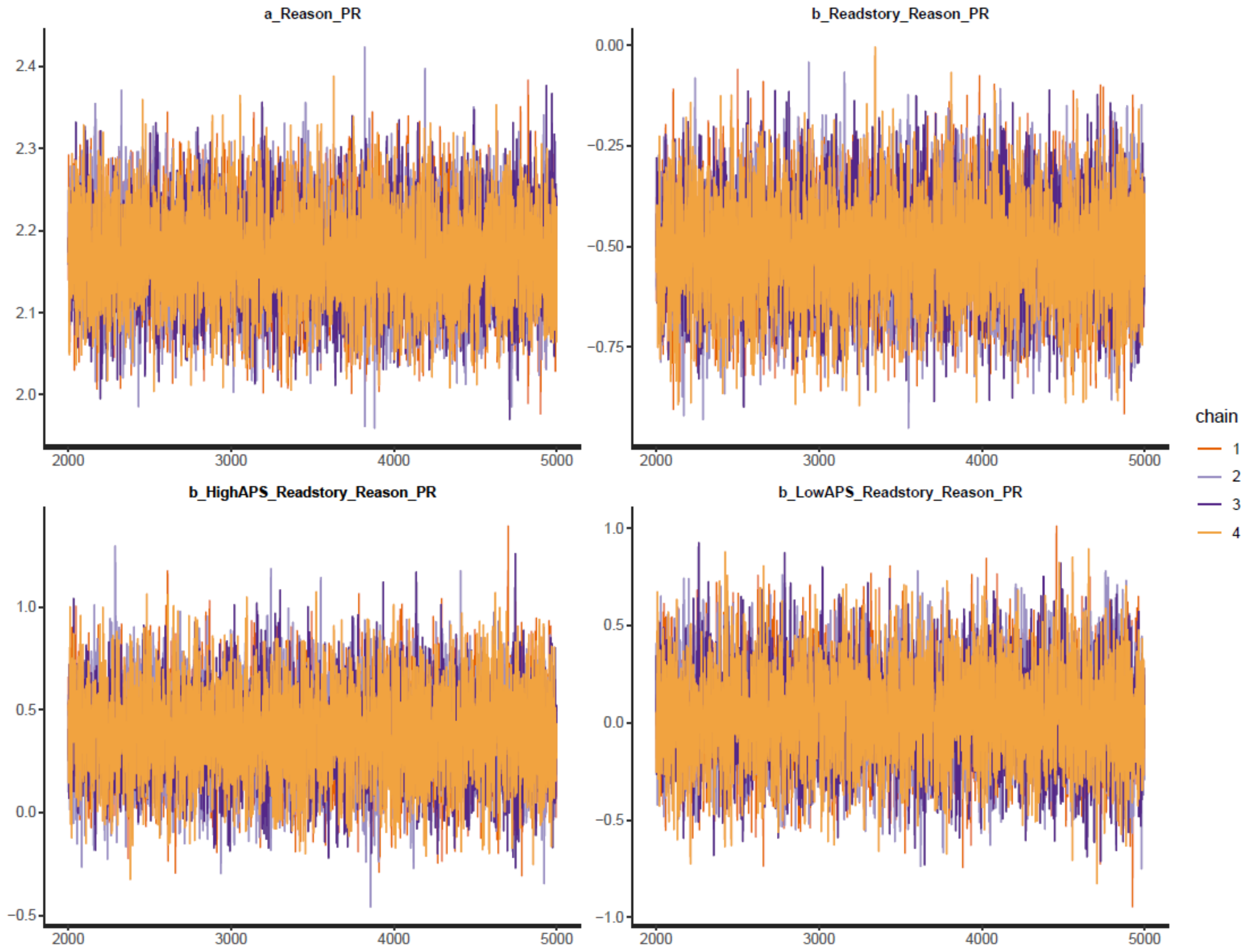

Figure S10: Trace plots for Model 2’s posterior coefficients

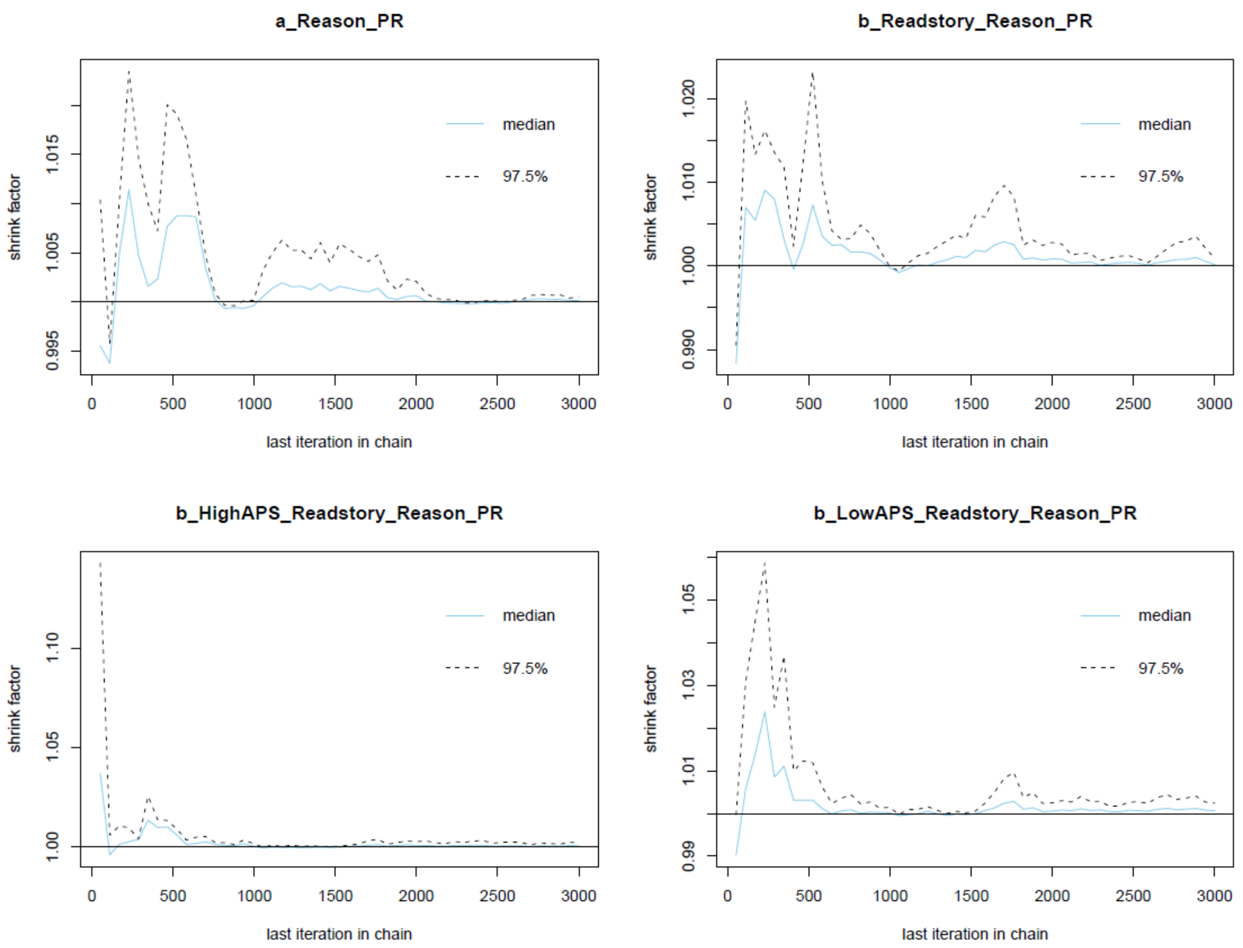

Figure S11: Gelman plots for Model 2’s posterior coefficients

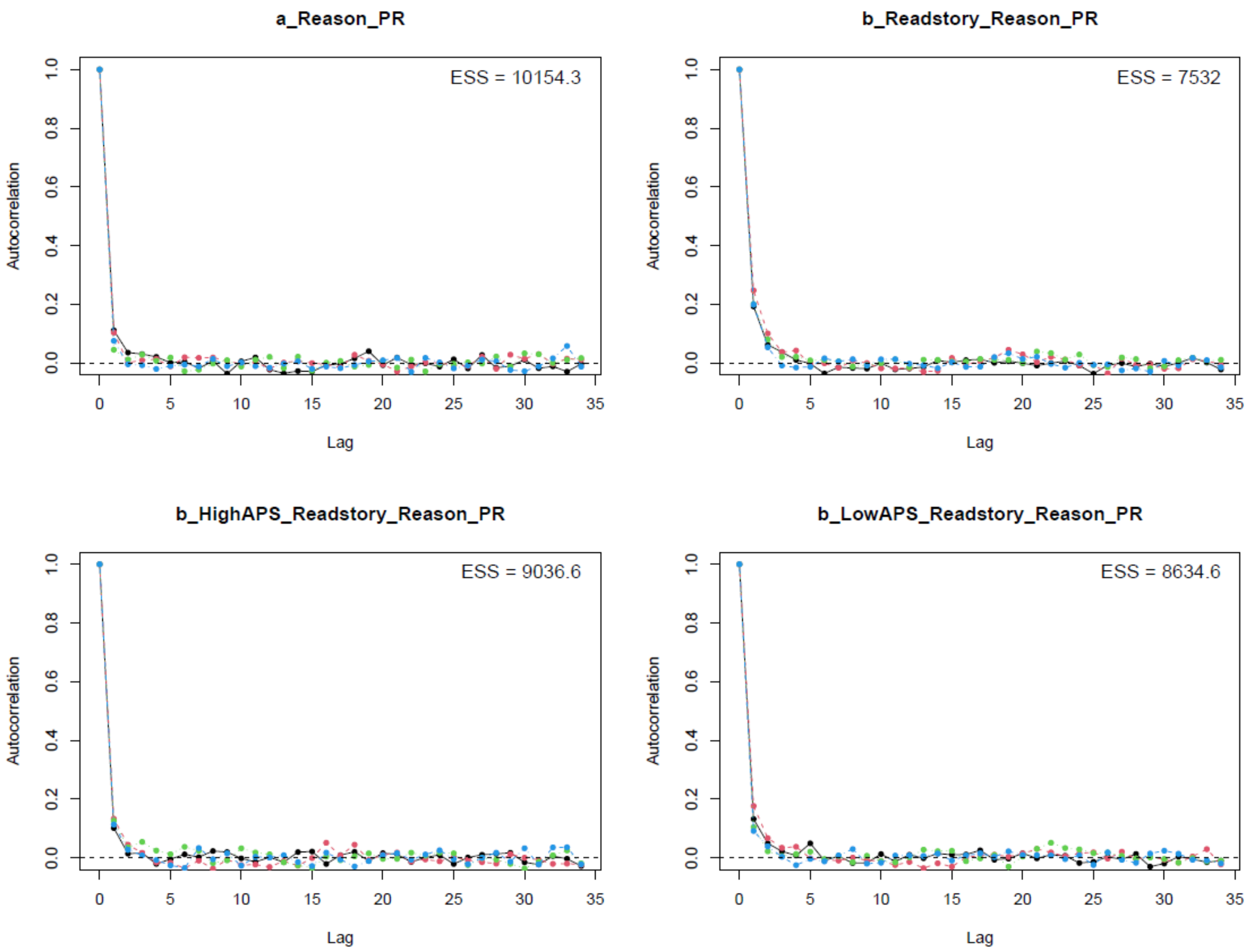

Figure S12: Autocorrelation plots for Model 2's posterior coefficients

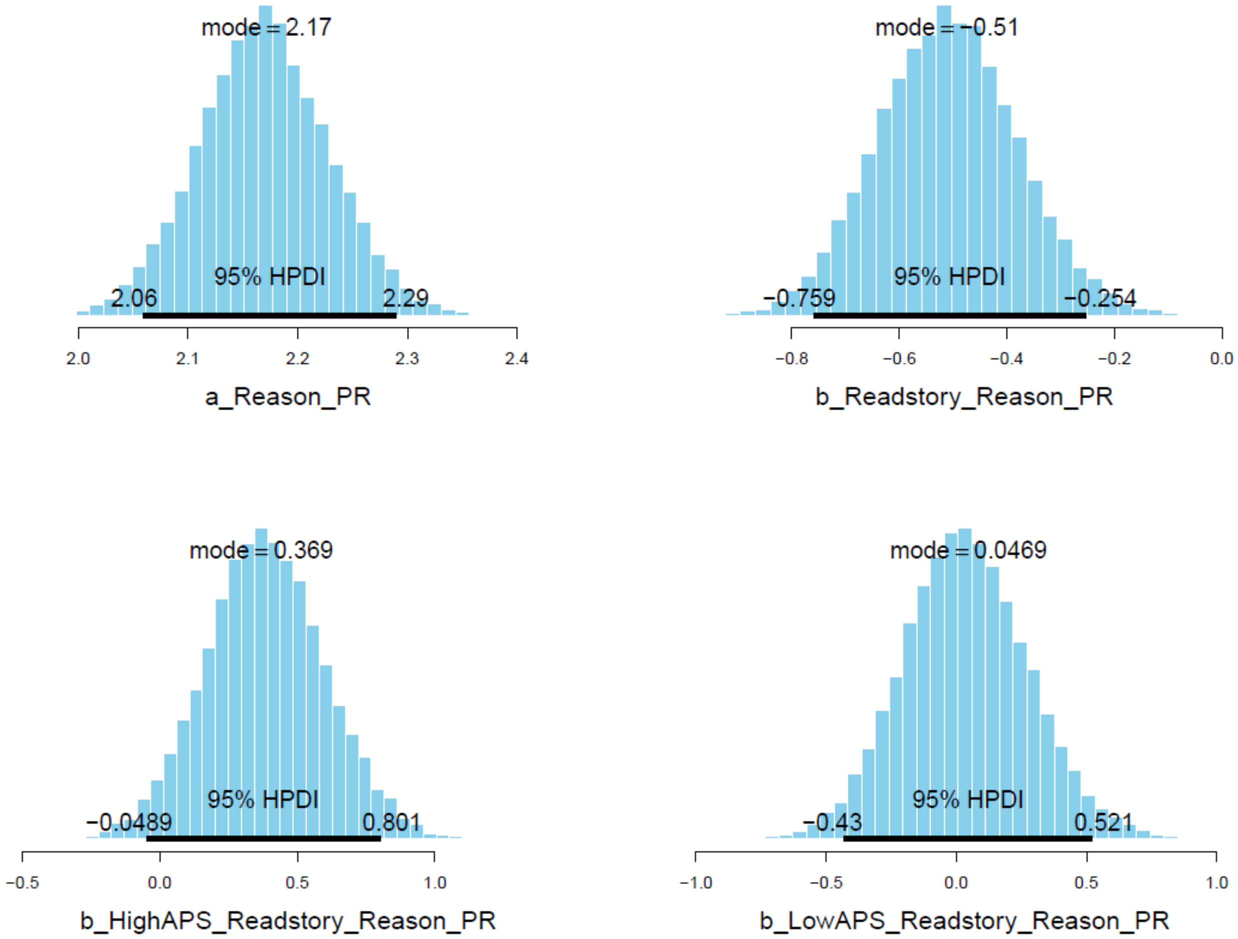

Figure S13: Distributions of Model 2’s posterior coefficients with HPDI at 95%

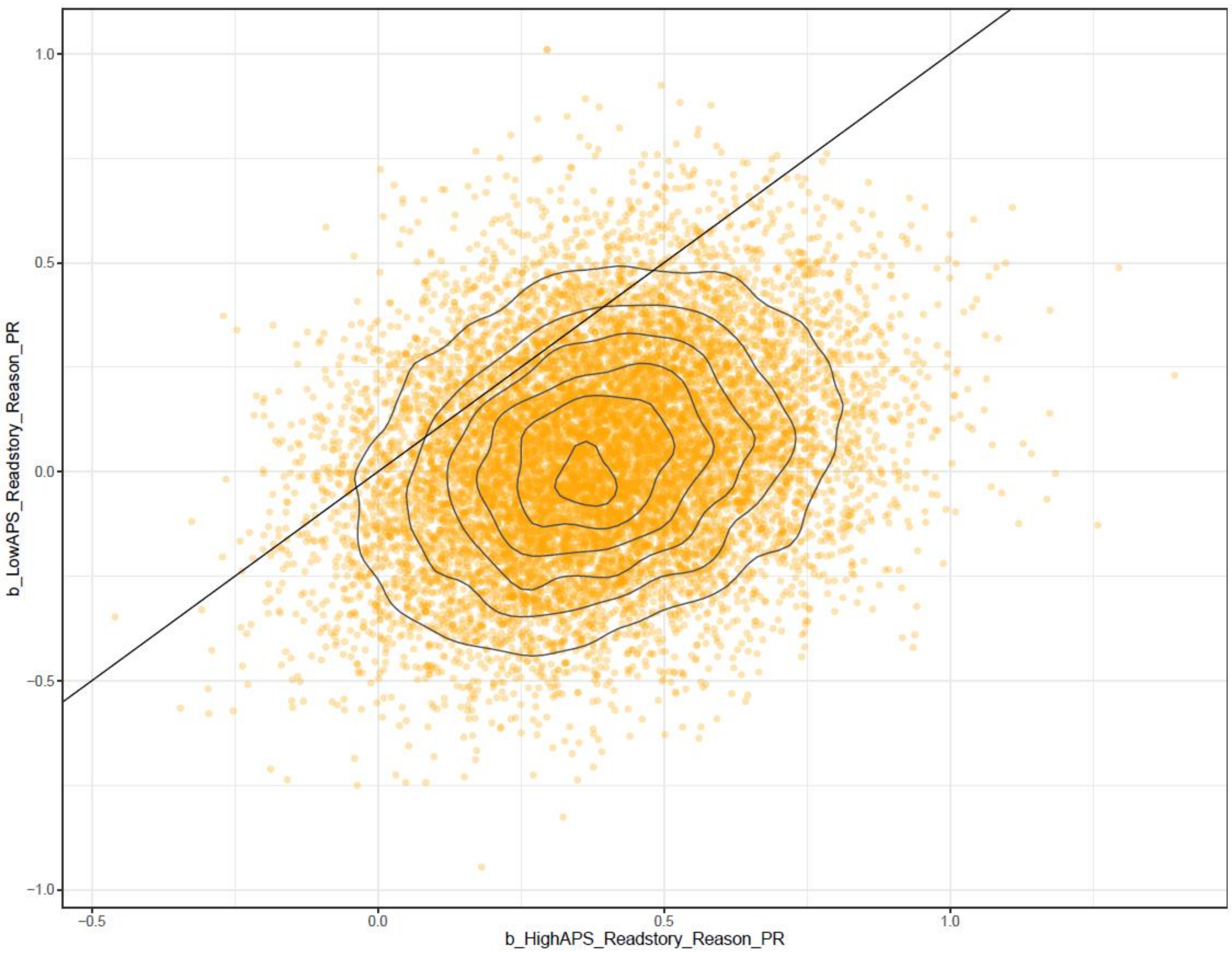

Figure S14: Pairwise distribution plot for model 2's *Readstory*HighAPS* and *Readstory*LowAPS*

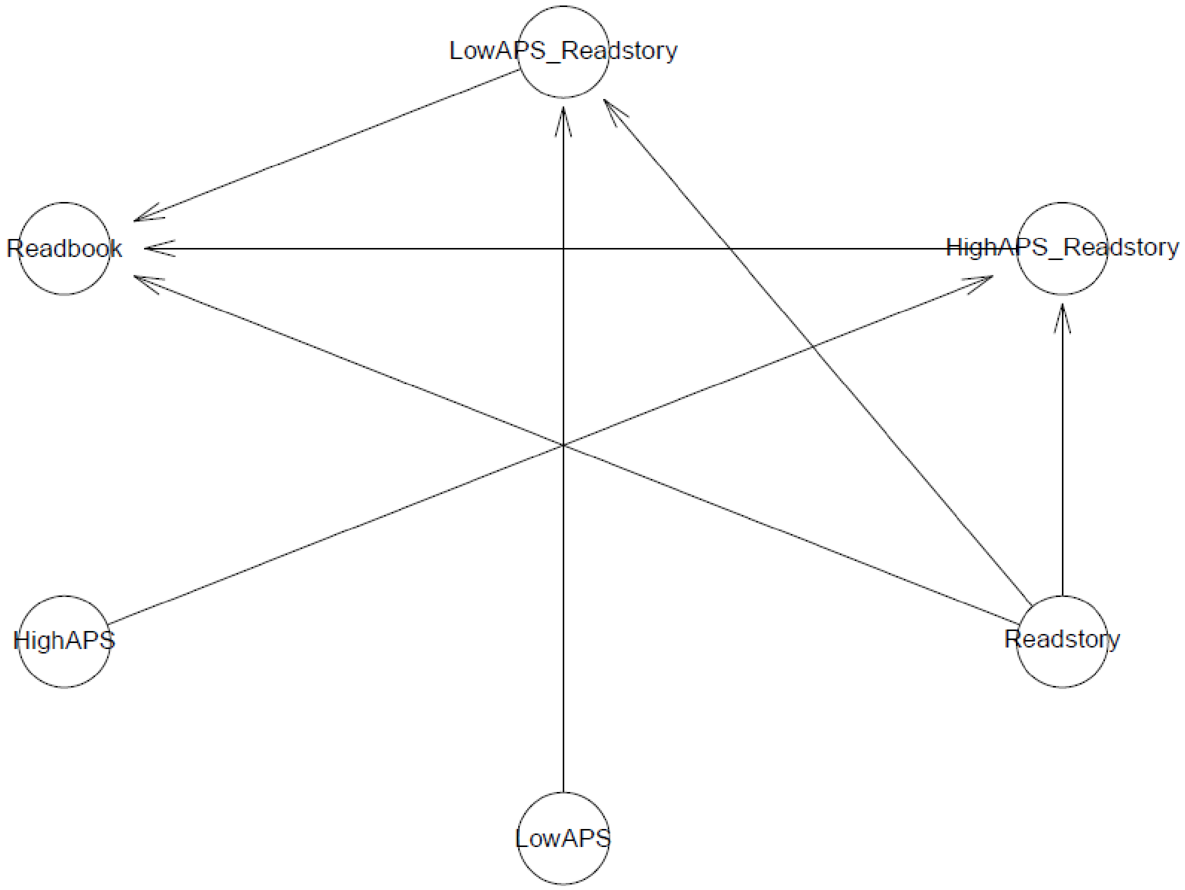

Figure S15: Model 3's logical network

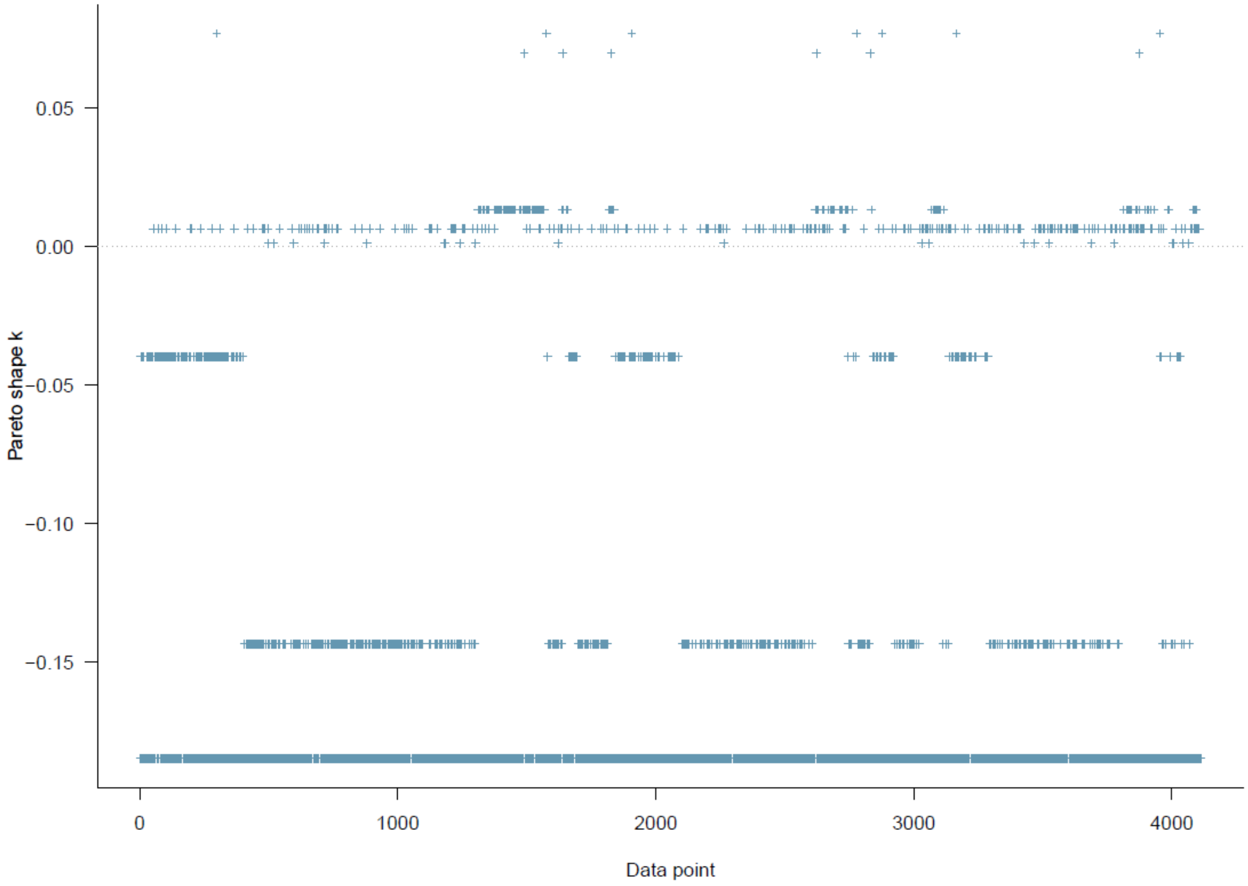

Figure S16: Model 3's PSIS diagnostic plot

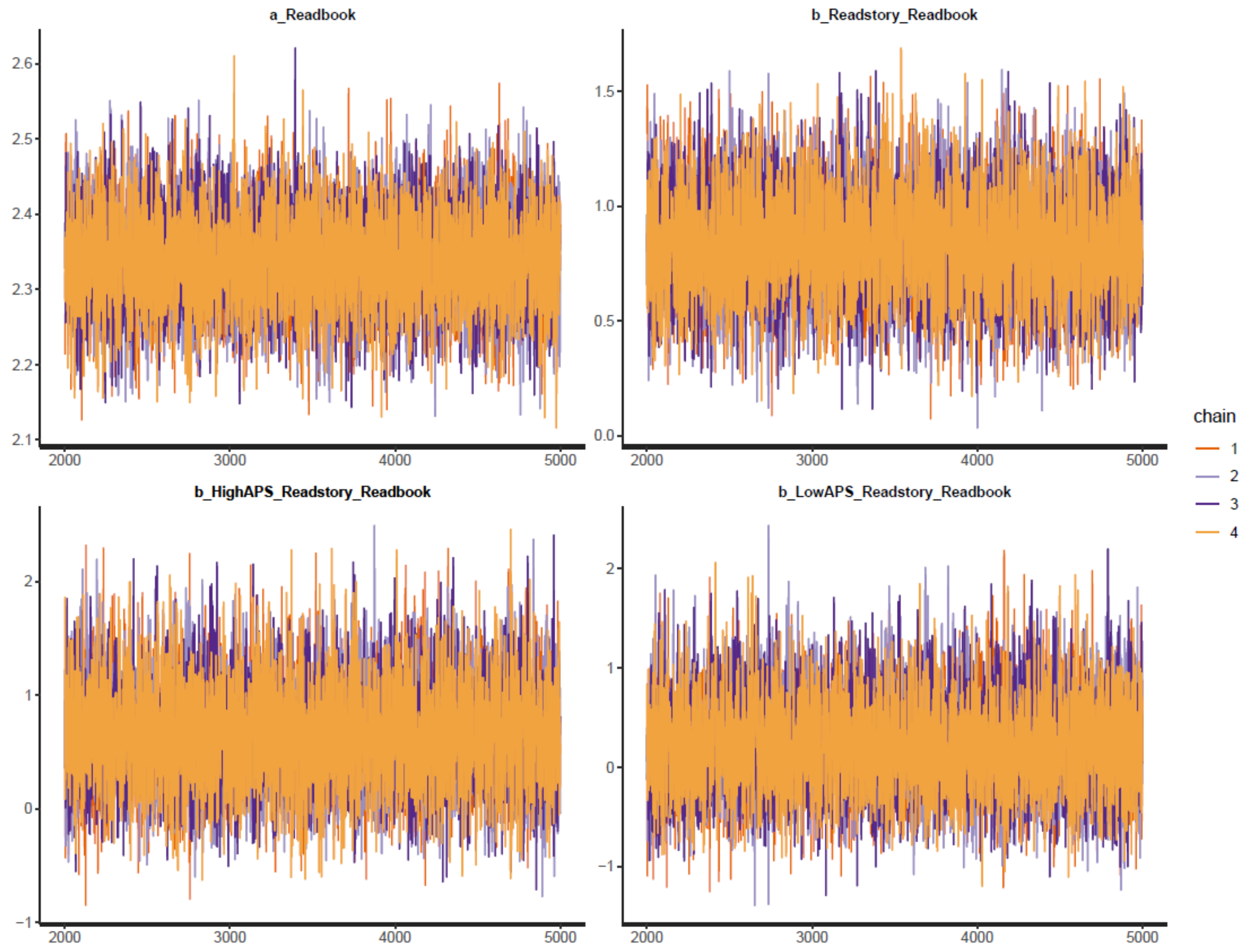

Figure S17: Trace plots for Model 3’s posterior coefficients

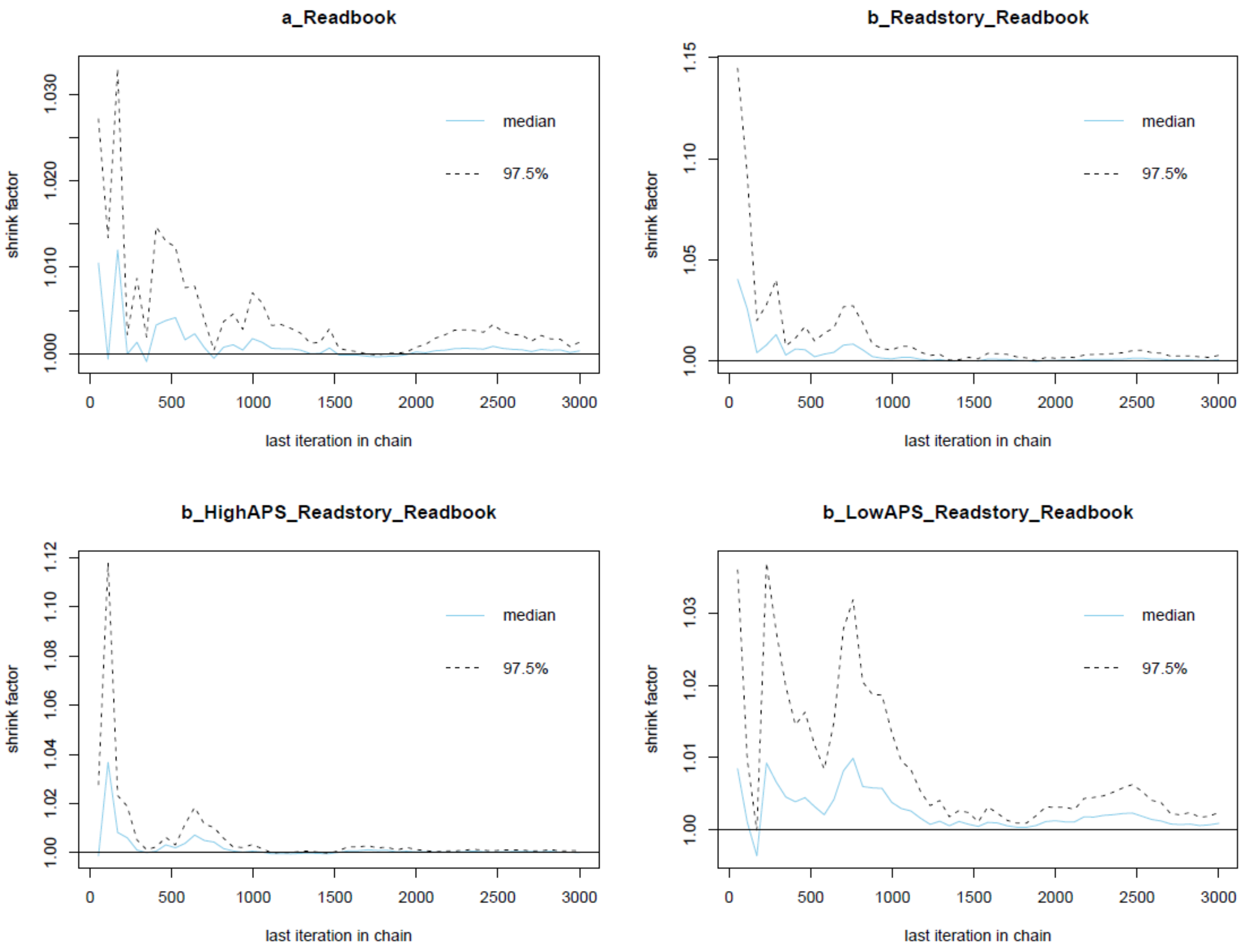

Figure S18: Gelman plots for Model 3's posterior coefficients

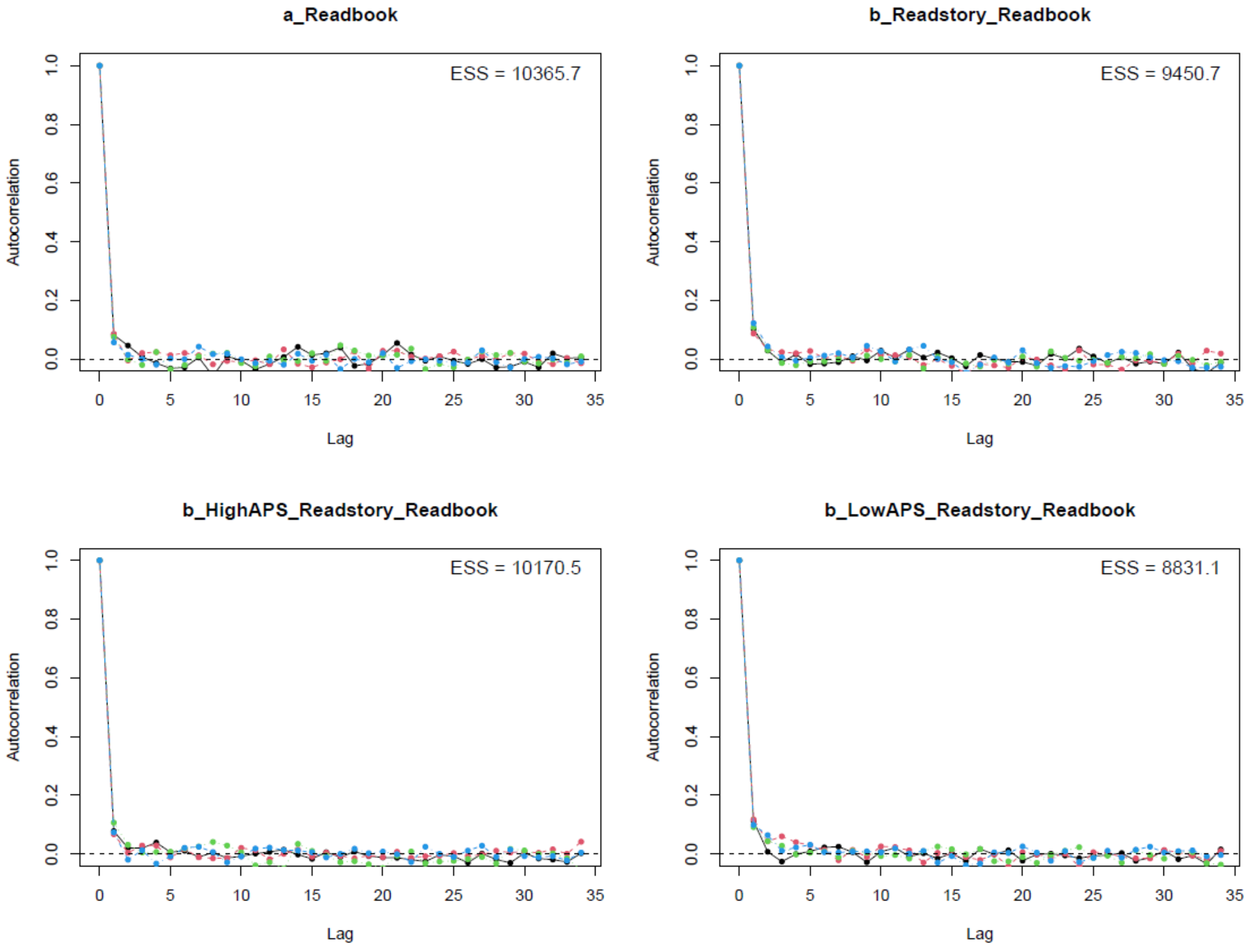

Figure S19: Autocorrelation plots for Model 3's posterior coefficients

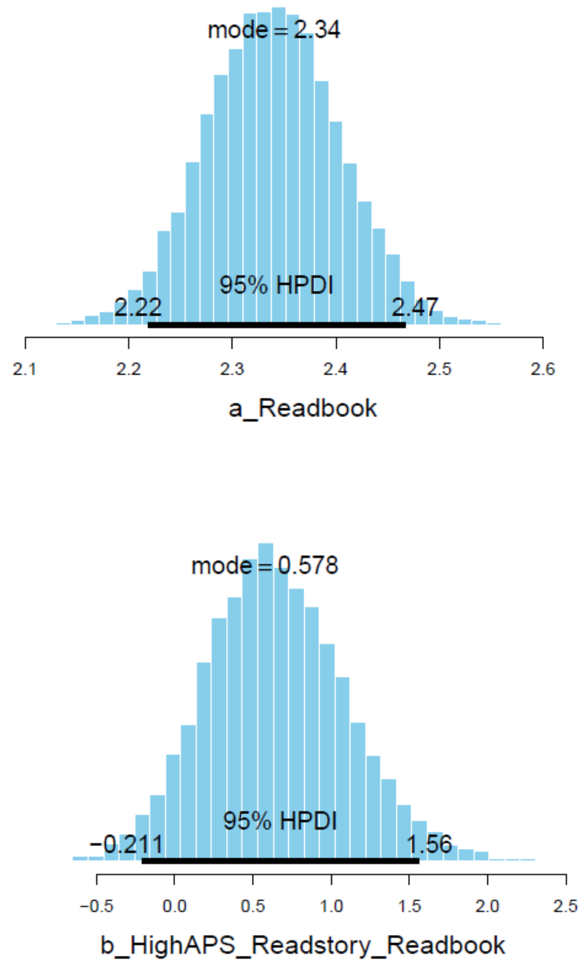

Figure S20: Distributions of Model 3's posterior coefficients with HPDI at 95%

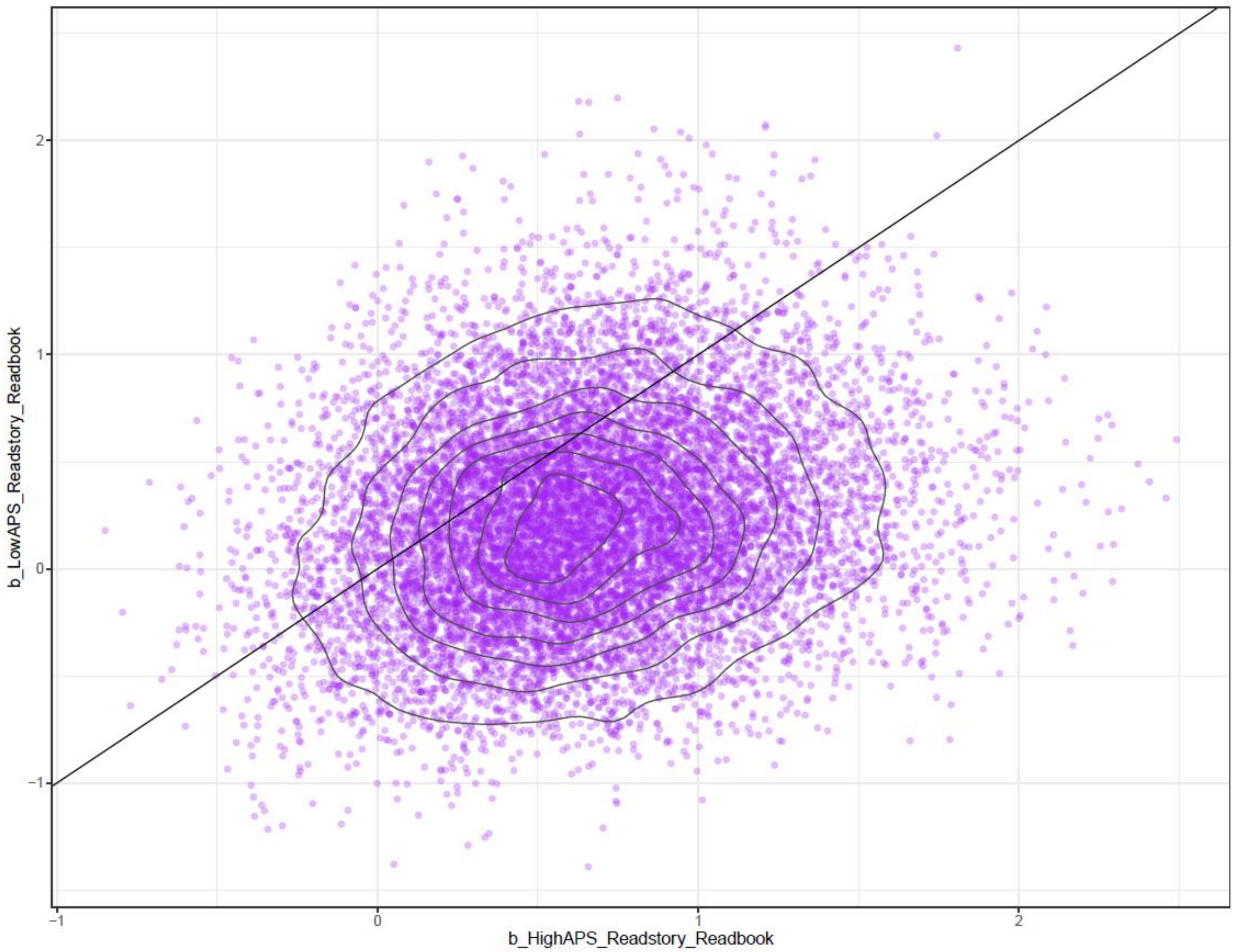

Figure S21: Pairwise distribution plot for model 3's *Readstory*HighAPS* and *Readstory*LowAPS*
