# Supplementary material for: Home Scholarly Culture, Book Selection Reason, and Academic Performance: Pathways to Book Reading Interest among Secondary School Students
Source: Eur J Investig Health Psychol Educ. 2021 Jun 1;11(2):468–95. doi: 10.3390/ejihpe11020034 (PMC8314380; doi:10.3390/ejihpe11020034)
Supplement: Supplementary file 1 [file ejihpe-11-00034-s001.zip › Codes for Pathways to reading interest.pdf]

Codes for “Home scholarly culture, book selection reason, and academic performance: Pathways to book reading interest among secondary school students”

Data preparation

```
data1 <- read.csv("C:/Users/…/SciData_STEM.csv",header = TRUE)
keeps <- c("HighAPS","LowAPS", "Readbook", "Reason_PR","Buybook","Readstory")
data1 <- data1[keeps]
data1<-na.omit(data1)
```

Package loading

```
library(bayesvl)
library(ggplot2)
library(cowplot)
```

Model 1

# Model construction

```
model1<-bayesvl()
model1<-bvl_addNode(model1,"Readbook","binom")
model1<-bvl_addNode(model1,"HighAPS","binom")
model1<-bvl_addNode(model1,"LowAPS","binom")
model1<-bvl_addNode(model1,"Reason_PR","binom")

model1<-bvl_addNode(model1,"HighAPS_Reason_PR","trans")
model1<-bvl_addNode(model1,"LowAPS_Reason_PR","trans")

model1<-bvl_addArc(model1,"HighAPS","HighAPS_Reason_PR","*")
model1<-bvl_addArc(model1,"Reason_PR","HighAPS_Reason_PR","*")

model1<-bvl_addArc(model1,"LowAPS","LowAPS_Reason_PR","*")
model1<-bvl_addArc(model1,"Reason_PR","LowAPS_Reason_PR","*")

model1<-bvl_addArc(model1,"Reason_PR","Readbook","slope")
model1<-bvl_addArc(model1,"HighAPS_Reason_PR","Readbook","slope")
model1<-bvl_addArc(model1,"LowAPS_Reason_PR","Readbook","slope")
```

# Stan code generation

```
model_string1 <- bvl_model2Stan(model1)
cat(model_string1)
```

Model Fitting

model1<-bvl_modelFit(model1, data1, warmup = 2000, iter = 5000, chains = 4,cores = 4)

Visualizations

- ***Figure 1***

bvl_bnPlot(model1)

- ***Figure 2***

loo1<-bvl_stanLoo(model1)
plot(loo1)

- ***Figure 3***

bvl_plotTrace(model1)

- ***Figure 4***

bvl_plotGelmans(model1,NULL, 2,2)

- ***Figure 5***

bvl_plotAcfs(model1,NULL, 2,2)

- ***Figure 6***

model1_figure6a<-bvl_plotIntervals(model1,c("b_Reason_PR_Readbook","b_HighAPS_Reason_PR_Readbook","b_LowAPS_Reason_PR_Readbook"))+theme_bw()

model1_figure6b<-bvl_plotDensity(model1,c("b_Reason_PR_Readbook","b_HighAPS_Reason_PR_Readbook","b_LowAPS_Reason_PR_Readbook"))+theme_bw()

plot_grid(model1_figure6a,model1_figure6b,nrow = 2,labels = c('A','B'),align = TRUE)

- ***Figure 7***

bvl_plotParams(model1,row=2,col=2,credMass = 0.95,params = NULL)

- ***Figure 8***

```
bvl_plotDensity2d(model1,"b_HighAPS_Reason_PR_Readbook","b_LowAPS_Reason_PR_Readbook",color_scheme = "red")+theme_bw()
```

Model 2
# Model construction

```
model2<-bayesvl()
model2<-bvl_addNode(model2,"Reason_PR","binom")
model2<-bvl_addNode(model2,"HighAPS","binom")
model2<-bvl_addNode(model2,"LowAPS","binom")
model2<-bvl_addNode(model2,"Readstory","binom")

model2<-bvl_addNode(model2,"HighAPS_Readstory","trans")
model2<-bvl_addNode(model2,"LowAPS_Readstory","trans")

model2<-bvl_addArc(model2,"HighAPS","HighAPS_Readstory","*")
model2<-bvl_addArc(model2,"Readstory","HighAPS_Readstory","*")

model2<-bvl_addArc(model2,"LowAPS","LowAPS_Readstory","*")
model2<-bvl_addArc(model2,"Readstory","LowAPS_Readstory","*")

model2<-bvl_addArc(model2,"Readstory","Reason_PR","slope")
model2<-bvl_addArc(model2,"HighAPS_Readstory","Reason_PR","slope")
model2<-bvl_addArc(model2,"LowAPS_Readstory","Reason_PR","slope")
```

# Stan code generation

```
model_string2 <- bvl_model2Stan(model2)
cat(model_string2)
```

# Model Fitting

```
model2<-bvl_modelFit(model2, data1, warmup = 2000, iter = 5000, chains = 4,cores = 4)
```

# Visualizations

- ***Figure 9***

```
bvl_bnPlot(model2)
```

- ***Figure 10***

```
loo2<-bvl_stanLoo(model2)
plot(loo2)
```

- ***Figure 11***

```
bvl_plotTrace(model2)
```

- ***Figure 12***

```
bvl_plotGelmans(model2,NULL, 2,2)
```

- ***Figure 13***

```
bvl_plotAcfs(model2,NULL, 2,2)
```

- ***Figure 14***

```
model2_figure14a<-
bvl_plotIntervals(model2,c("b_Readstory_Reason_PR","b_HighAPS_Readstory_Reason_PR","b_LowAPS_Readstory_Reason_PR"))+theme_bw()

model2_figure14b<-
bvl_plotDensity(model2,c("b_Readstory_Reason_PR","b_HighAPS_Readstory_Reason_PR","b_LowAPS_Readstory_Reason_PR"))+theme_bw()

plot_grid(model2_figure14a, model2_figure14b,nrow = 2, labels = c('A', 'B'),align = TRUE)
```

- ***Figure 15***

```
bvl_plotParams(model2,row=2,col=2,credMass = 0.95,params = NULL)
```

- ***Figure 16***

```
bvl_plotDensity2d(model2,"b_HighAPS_Readstory_Reason_PR","b_LowAPS_Readstory_Reason_PR",color_scheme = "orange")+theme_bw()
```

Model 3
# Model construction

```
model3<-bayesvl()
model3<-bvl_addNode(model3,"Readbook","binom")
model3<-bvl_addNode(model3,"HighAPS","binom")
```

```
model3<-bvl_addNode(model3,"LowAPS","binom")
model3<-bvl_addNode(model3,"Readstory","binom")

model3<-bvl_addNode(model3,"HighAPS_Readstory","trans")
model3<-bvl_addNode(model3,"LowAPS_Readstory","trans")

model3<-bvl_addArc(model3,"HighAPS","HighAPS_Readstory","*")
model3<-bvl_addArc(model3,"Readstory","HighAPS_Readstory","*")

model3<-bvl_addArc(model3,"LowAPS","LowAPS_Readstory","*")
model3<-bvl_addArc(model3,"Readstory","LowAPS_Readstory","*")

model3<-bvl_addArc(model3,"Readstory","Readbook","slope")
model3<-bvl_addArc(model3,"HighAPS_Readstory","Readbook","slope")
model3<-bvl_addArc(model3,"LowAPS_Readstory","Readbook","slope")
```

Stan code generation

```
model_string3 <- bvl_model2Stan(model3)
cat(model_string3)
```

Model Fitting

```
model3<-bvl_modelFit(model3, data1, warmup = 2000, iter = 5000, chains = 4,cores = 4)
```

Visualizations

- ***Figure 17***

```
bvl_bnPlot(model3)
```

- ***Figure 18***

```
loo3<-bvl_stanLoo(model3)
plot(loo3)
```

- ***Figure 19***

```
bvl_plotTrace(model3)
```

- ***Figure 20***

```
bvl_plotGelmans(model3,NULL, 2,2)
```

- ***Figure 21***

```
bvl_plotAcfs(model3,NULL, 2,2)
```

- ***Figure 22***

```
model3_figure22a<-
bvl_plotIntervals(model3,c("b_Readstory_Readbook","b_HighAPS_Readstory_Readbook","b_LowAPS_Readstory_Readbook"))+theme_bw()

model3_figure22b<-
bvl_plotDensity(model3,c("b_Readstory_Readbook","b_HighAPS_Readstory_Readbook","b_LowAPS_Readstory_Readbook"))+theme_bw()

plot_grid(model3_figure22a, model3_figure22b,nrow = 2,ncol = 1, labels = c('A', 'B'),align = TRUE)
```

- ***Figure 23***

```
bvl_plotParams(model3,row=2,col=2,credMass = 0.95,params = NULL)
```

- ***Figure 24***

```
bvl_plotDensity2d(model3,"b_HighAPS_Readstory_Readbook","b_LowAPS_Readstory_Readbook",color_scheme = "purple")+theme_bw()
```
